# Neutrophils-related host factors associated with severe disease and fatality in patients with influenza infection

**DOI:** 10.1038/s41467-019-11249-y

**Published:** 2019-07-31

**Authors:** Benjamin M. Tang, Maryam Shojaei, Sally Teoh, Adrienne Meyers, John Ho, T. Blake Ball, Yoav Keynan, Amarnath Pisipati, Aseem Kumar, Damon P. Eisen, Kevin Lai, Mark Gillett, Rahul Santram, Robert Geffers, Jens Schreiber, Khyobeni Mozhui, Stephen Huang, Grant P. Parnell, Marek Nalos, Monika Holubova, Tracy Chew, David Booth, Anand Kumar, Anthony McLean, Klaus Schughart

**Affiliations:** 1 Department of Intensive Care Medicine, Nepean Hospital, Sydney, Australia; 2 Centre for Immunology and Allergy Research, The Westmead Institute for Medical Research, Sydney, Australia; 3 Respiratory Tract Infection Research Node, Marie Bashir Institute for Infectious Diseases and Biosecurity, Sydney, Australia; 4 National HIV and Retrovirology Laboratories, JC Wilt Infectious Disease Research Centre, Public Health Agency of Canada, Department of Medical Microbiology and Infectious Disease, University of Manitoba, Winnipeg, Canada; 5 Department of Internal Medicine, Medical Microbiology and Community Health Sciences, University of Manitoba, Winnipeg, Canada; 6 Department of Chemistry and Biological Chemistry, Harvard University, Cambridge, MA USA; 7 Department of Chemistry and Biochemistry, Laurentian University, Laurentian, Canada; 8 Townsville Hospital, Townsville, Queensland Australia; 9 Department of Emergency Medicine, Westmead Hospital, Sydney, Australia; 10 Department of Emergency Medicine, Royal North Shore Hospital, Sydney, Australia; 11 Department of Emergency Medicine, St. Vincent Hospital, Sydney, Australia; 12 Genome Analytics, Helmholtz Centre for Infection Research, Braunschweig, Germany; 13 Otto-von-Guerike University of Magdeburg, Clinic of Pneumology, Magdeburg, Germany; 14 Department of Preventive Medicine, University of Tennessee Health Science Centre, Memphis, TN USA; 15 Department of Internal Medicine, Medical Faculty Plzen, Charles University Prague, Staré Město, Czech Republic; 16 Biomedical Centre, Medical Faculty Plzen, Charles University Prague, Staré Město, Czech Republic; 17 Sydney Informatic Hub, The University of Sydney, Sydney, Australia; 18 Section of Critical Care Medicine and Section of Infectious Diseases, Departments of Medicine, Medical Microbiology and Pharmacology, University of Manitoba, Winnipeg, Canada; 19 Department of Infection Genetics, Helmholtz Centre for Infection Research, Braunschweig, Germany; 20 University of Veterinary Medicine, Hannover, Germany; 21 Department of Microbiology, Immunology and Biochemistry, University of Tennessee Health Science Centre, Memphis, TN USA

**Keywords:** Infectious-disease diagnostics, Viral infection, Respiratory tract diseases, Pathogenesis, Immunopathogenesis

## Abstract

Severe influenza infection has no effective treatment available. One of the key barriers to developing host-directed therapy is a lack of reliable prognostic factors needed to guide such therapy. Here, we use a network analysis approach to identify host factors associated with severe influenza and fatal outcome. In influenza patients with moderate-to-severe diseases, we uncover a complex landscape of immunological pathways, with the main changes occurring in pathways related to circulating neutrophils. Patients with severe disease display excessive neutrophil extracellular traps formation, neutrophil-inflammation and delayed apoptosis, all of which have been associated with fatal outcome in animal models. Excessive neutrophil activation correlates with worsening oxygenation impairment and predicted fatal outcome (AUROC 0.817–0.898). These findings provide new evidence that neutrophil-dominated host response is associated with poor outcomes. Measuring neutrophil-related changes may improve risk stratification and patient selection, a critical first step in developing host-directed immune therapy.

## Abstract (web summary)

Identification of host factors associated with severe influenza infection could provide insights into treatment options. Here, the authors provide transcriptomic analyses of blood from >100 influenza infected patients and show that changes in circulating neutrophils are associated with severe influenza infection.

## Introduction

Influenza is one of the most prevalent respiratory virus infections in the world, with one out of five people suffering from it annually^1^. Severe influenza, the most serious form of influenza infection, is characterized by lung inflammation, refractory hypoxaemia, and respiratory failure. The change from a mild/moderate, self-limited “flu” illness toward severe influenza represents a critical transition point in the clinical course of influenza infection. The initial mild “flu” begins in the upper airway, where tissue damage is minimal and the infection is well controlled by an effective, local immune response. The spread of infection to the lower airway is accompanied by a dysregulated local and systematic immune response (both innate and adaptive immunity)^2–10^. The spread occurs rapidly (within days) resulting in airway inflammation and extensive alveolar damage. When fully developed, severe influenza leads to significant respiratory complications, such as acute respiratory distress syndrome or secondary bacterial pneumonia. Mortality of severe influenza is high, ranging from to 10% (seasonal influenza) to 65% (in pandemic H1N1 patients needing extracorporeal membrane oxygenation support)^11,12^. Early antiviral treatment may improve the outcome^13^. However, mortality remains high in severe cases because the influenza-related lung inflammation is driven primarily by immunopathology^2–10^.

Host-directed therapy offers a promising new approach to target immunopathology and may reduce the mortality of severe influenza^14,15^. However, a myriad of immunological abnormalities have been identified in severe influenza and it is unclear which of these should be targeted by host-directed therapy^9,16^. To identify host factors associated with severe disease, a unifying framework of influenza host response is needed. A rational approach to developing such framework is to use a network analysis approach to identify all disease-relevant host factors and rank these factors in the order of their relative contribution to immunopathology/outcomes^17^. The top-ranking host factors may then be targeted by using host-directed therapy—as has been successfully demonstrated in HIV medicine^18^.

Gene-expression analysis provides a unifying framework in understanding the host factors associated with influenza pathogenesis. A common approach is to measure gene expression of circulating leukocytes in infected patients^19–21^. Several recent studies have adopted this approach, but these studies were limited by including only asymptomatic individuals^21^, patients with mild illness^19^ or those presenting in early stage of the disease^20^. In this study, we use a network analysis approach to analyze the host response of circulating leukocytes in a large cohort of moderate-to-severe influenza patients. Using weighted gene co-expression network analysis, we identify host factors that were statistically significantly associated with severe influenza infection. We then quantify each factor’s contribution to infection by ranking them based on their strength of association with disease severity. Here, we report that this approach produces a global overview of the immunological landscape of the influenza infection and enable us to identify several key biological processes that underpin the development of severe influenza and fatality.

## Results

### Patient characteristics

**Fig. 1:**
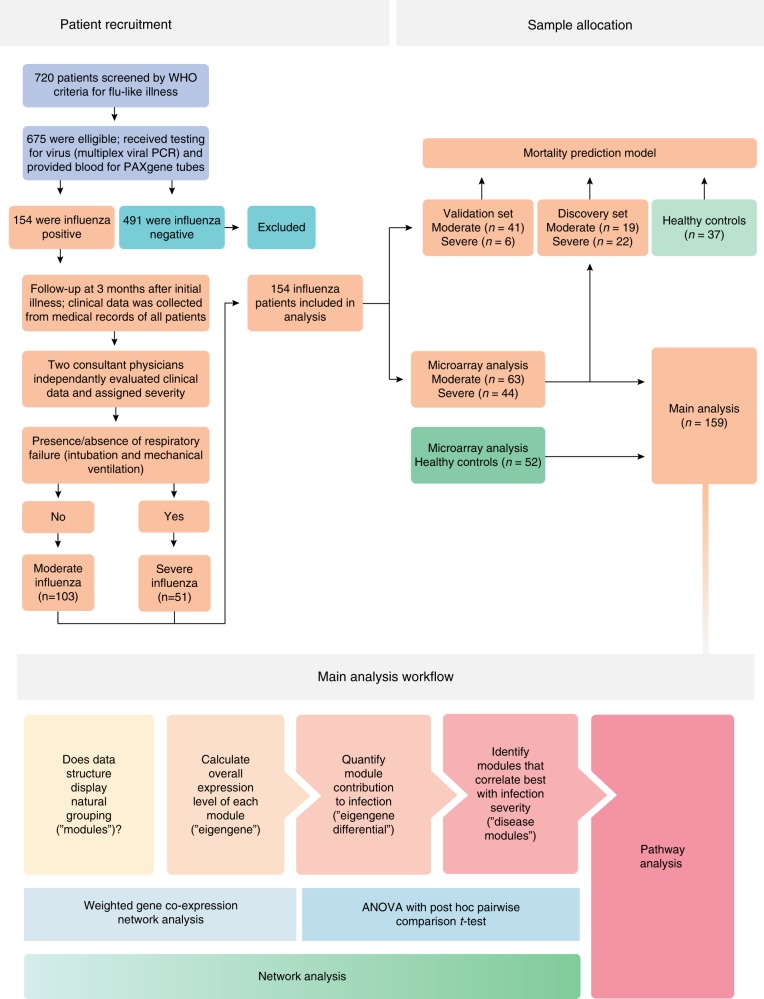
Study scheme. Flow chart shows patient recruitment, sample allocation, and analysis workflow

In this prospective study, we screened 720 patients who met the World Health Organization’s criteria for influenza-like illness. After initial evaluation, 154 patients had clinical and virological evidence of influenza infection. Of the 154 collected blood samples, 107 samples were randomly selected for microarray analysis; the remaining 47 samples were reserved for a subsequent validation analysis (Fig. 1).

Of the 107 samples assigned to microarray analysis, 63 were from patients with moderate infection who did not receive mechanical ventilation (referred as the “moderate group”) and 44 from patients with severe infection who developed respiratory failure requiring mechanical ventilation (referred as the “severe group”). Samples from healthy donors were also included (*n* = 52).

Initial analyses showed that patients in the severe group were more likely to have received antiviral treatment (*p* < 0.0001) and to have developed respiratory failure (*p* < 0.0001) (Table 1). The severe group also had a much higher morbidity/mortality (e.g., organ failure, length of stay, and mortality) compared to the moderate group. However, other than infection severity, the moderate and severe groups did not differ significantly in factors that might affect the gene-expression of host response, including age, gender, pre-existing illnesses, duration of symptoms, or the types of virus strains. (Table 1).

**Table 1:** Demographic and clinical characteristics of the study groups

	Healthy controls	Moderate	Severe	*p* Values^a^
Number	52	63	44	
Gender (males/females)	19/33	27/36	17/27	0.69
Age/years (mean + SD)	43.5 (14.5)	52.6 (19)	46.5 (16)	0.11
Duration of onset		4.0 days	5.6 days	0.19
*Symptoms*				
Cough		25 (40%)	37 (84%)	<0.0001
Fever/chills		16 (25%)	32 (73%)	<0.0001
Dyspnea		14 (22%)	44 (100%)	<0.0001
Malaise/aches		22 (35%)	28 (64%)	<0.0001
*Pre-existing illnesses*				
Asthma		8 (13%)	9 (20%)	0.25
Chronic lung disease		7 (11%)	9 (20%)	0.12
Ischemic heart disease		12 (19%)	6 (14%)	0.45
Hypertension/previous CVA		11 (17%)	5 (11%)	0.31
Diabetes		8 (13%)	9 (20%)	0.25
Cancer/on chemotherapy		5 (7.9%)	2 (4.5%)	0.57
*Virology*				
Positive influenza PCR		63 (100%)	44 (100%)	0.99
Influenza A subtype		53 (84%)	40 (91%)	0.19
Influenza B subtype		10 (16%)	4 (9%)	0.19
Antiviral treatment (Tamiflu)		3 (5%)	40 (91%)	<0.0001
*Respiratory support*				
Mechanical ventilation		0 (0%)	42 (95%)	<0.0001
Noninvasive support (CPAP)		0 (0%)	2 (5%)	<0.0001
*Secondary complications*				
Bacterial pneumonia		0 (0%)	7 (16%)	<0.0001
Shock		0 (0%)	12 (27%)	<0.0001
Acute renal failure		1 (1.6%)	6 (14%)	0.0006
Multiple organ failure		0 (0%)	14 (32%)	<0.0001
*Outcomes*				
Hospitalization		45 (71%)	44 (100%)	<0.0001
Admission to ICU		7 (11%)	44 (100%)	<0.0001
Length of ICU stay		3.5 days	18.7 days	0.015
Length of hospital stay		1.4 days	26 days	<0.0001
Death		0 (0%)	9 (20%)	<0.0001

^a^*p* Values are calculated by comparing moderate and severe groups using Mann–Whitney test for continuous variables or chi-square test for categorical variables. ICU denotes intensive care unit

### Host response in moderate and severe influenza infection

**Fig. 2:**
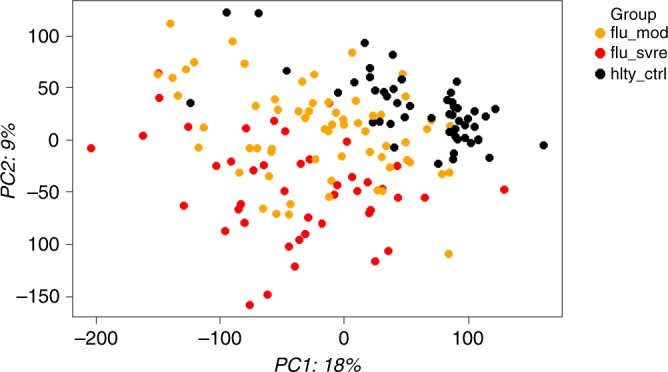
Principal component analysis (PCA). Unsupervised principal component analysis was performed across all samples (including healthy controls, moderate, and severe groups) using normalized log_2_ gene-expression levels. Based on two principal components (PC1 and PC2), moderate influenza (yellow data points) and severe influenza (red data points) showed evidence of separation in gene-expression space. The source data are available in Gene Expression Omnibus (GSE 101702)

We applied principal component analysis (PCA) to the blood transcriptome data to visualize the overall trend in gene-expression values across all samples. This preliminary examination suggested that gene-expression differed between phenotypes (healthy controls, moderate, and severe) (Fig. 2). We then assessed whether this difference could be due to cell count variations between these groups, since gene-expression difference may be attributed to altered cell counts, altered transcripts per cell or both. This analysis showed that the cell count did not account for the observed difference in gene-expression since leukocytes in both groups were within normal ranges: moderate (8.1 × 1000/mm^3^) and severe (9.6 × 1000/mm^3^). Furthermore, leukocyte counts between these two groups were not significantly different (*p* = 0.76) (Supplementary Fig. 1).

Since peripheral blood consists of many cell subsets (e.g., dendritic cells, B cells, monocytes, and CD4/CD8 lymphocytes), the different cell compositions between moderate and severe groups might also account for the observed gene-expression difference. The cell subsets were not routinely measured in clinical practice and thus were not available for analysis in this study. To circumvent this problem, we decomposed the peripheral blood transcriptome into relative abundances of cell subsets by measuring gene-expression markers of 33 cell subsets (using digital cell quantification). This analysis showed that there were no differences in the relative abundance of cells subsets between the moderate and severe groups, with the exception of pro-erythroblasts (Supplementary Fig. 2). This finding, together with no significant difference in baseline characteristics or premorbid comorbidities (between the moderate and severe groups) as noted earlier, indicate that the observed gene-expression differences in PCA were mainly due to altered cellular states associated with infection severity, rather than with cell numbers, cellular subset composition, or patient-related factors.

### Network analysis revealed five different biological themes

**Fig. 3:**
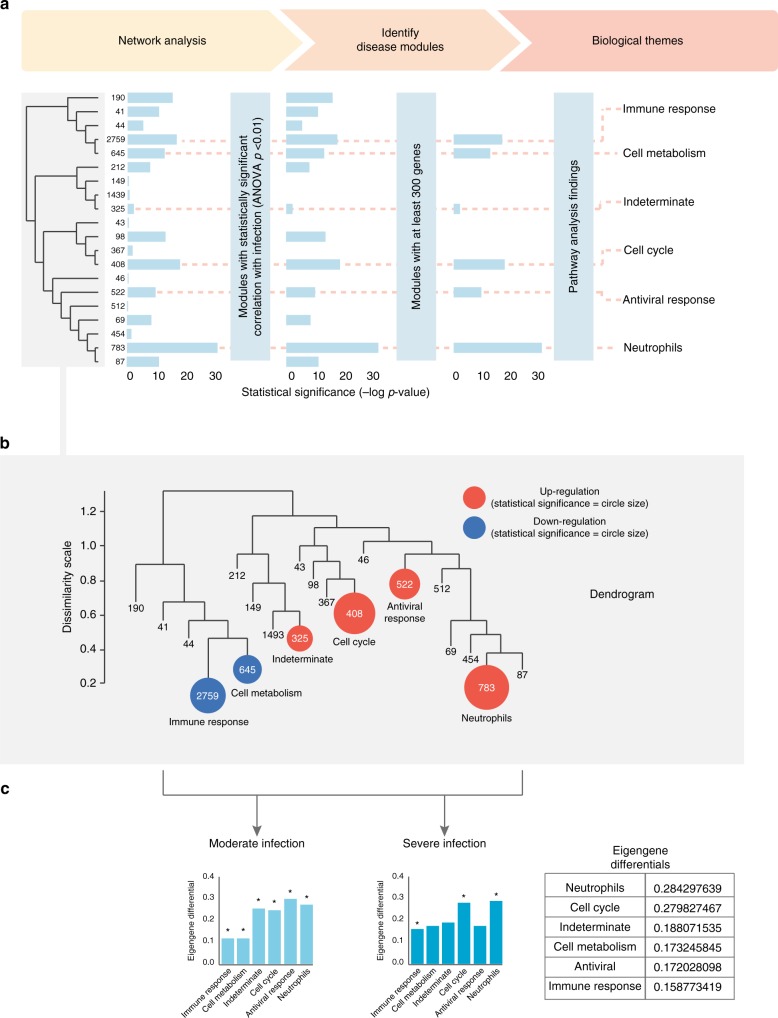
Main modules of host response to influenza infection. **a** Overview of main study findings. Weighted gene co-expression network analysis identified 20 modules. Horizontal bars represent statistical significance of eigengene values in each module, with disease severity as a factor in the ANOVA model. Dendrogram (left and also magnified below) shows gene numbers in each module. Six modules were kept because they were statistically significant in ANOVA model (*p* < 0.01) and met minimal gene number criteria (>300) for further analysis. Biological themes of these six modules are shown (right); one module did not show clear biological theme (hence named “Indeterminate”). **b** Gene-expression space in the dendrogram (based on an enlarged version of the dendrogram in above left). The statistically significant modules (*n* = 6) are highlighted in color; their number of genes are indicated by numbers inside the circles (circle sizes are proportional to statistical significance). The nonstatistically significant modules (*n* = 14) are shown as branches without circles; their number of genes are given at the foot of each branch. Gene-expression distance is indicated by (1) location in the dendrogram tree (modules within the same branch are more similar than those from different branches) and (2) dissimilarity scale (left-hand side of the dendrogram), which indicates the degree of gene connectivity between modules. **c** (Left) Eigengene differentials of moderate infection: eigengene differentials in each module represent the differences of eigengene mean expression levels between moderate infection and healthy controls (graphed with an offset of 0.2). (Right) Eigengene differentials of severe infection: eigengene differentials in each module represent the differences of eigengene mean expression levels between severe and moderate infection (graphed with an offset of 0.2). Stars above columns indicate statistically significant differences (by pairwise *t* test) between the groups. The actual levels of eigengene differentials between severe and moderate infection are also shown in an inserted table on the right. The source data are available in Gene Expression Omnibus (GSE 101702)

We next performed weighted gene co-expression network analysis (WGCNA) to identify disease modules associated with infection severity (Fig. 1). The WGCNA approach takes into consideration the inter-relationship between/within gene pathways, thus allowing us to quantify the complex interplay between cellular networks^22^. This analysis revealed 20 gene-expression modules (Fig. 3a). Of these 20 modules, 13 were statistically significant when correlated with infection status. Of these 13 modules, 6 modules contained more than 300 genes, which represented an adequate number of genes suitable for further analysis.

**Fig. 4:**
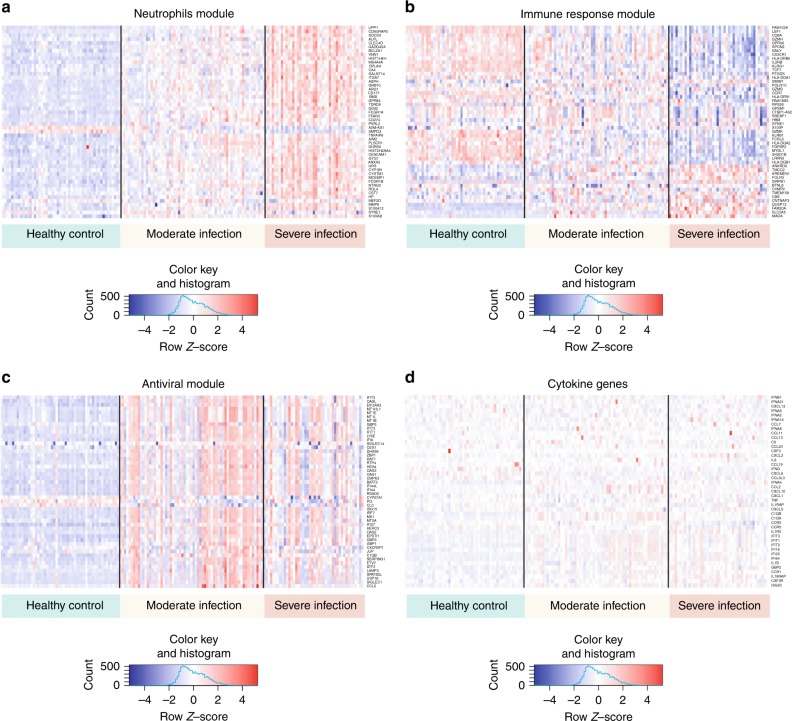
Neutrophils, immune response, antiviral modules, and cytokines heat maps. **a** Neutrophils module. **b** Immune response module. **c** Antiviral module. **d** Cytokine genes. Genes were sorted according to the calculated variance (high to low) and were clustered according to similarity in expression levels. Patients were grouped into control, moderate, and severe groups. The top 50 genes with the highest variance are presented in these heat maps. The source data are available in Gene Expression Omnibus (GSE 101702)

We noted that these six modules were well separated in gene-expression space (Fig. 3b), which suggested that the influenza host response is driven by multiple host factors, rather than a single dominant process. Further examination of the genes within these modules indicated that the modules corresponded broadly to five different biological themes (antiviral response, immune response, cell cycle, neutrophils, and cell metabolism) (Fig. 4). One module, the indeterminate module, did not display a discernible biological theme. Within each module, multiple cell types were represented (e.g., lymphocytes, monocytes, and natural killer cells). However, the neutrophils module was a notable exception because most genes were neutrophil-related (only a few genes were related to non-neutrophils leukocytes).

We asked whether the biological themes observed in these modules could be related to time-dependent changes in blood composition during infection. Influenza infection is known to be associated with a relative lymphopenia/neutrophilia, caused by a leukocyte redistribution between blood and peripheral compartments (e.g., bone marrow). This redistribution is usually transient and appears only during the first 2 days of influenza infection ^23^. For most patients in this study, collection of blood samples occurred between day 4 and day 6 of infection (Table 1), thus making it unlikely that the observed biological themes were related to leukocyte redistribution. Indeed, leukocyte differentials in these patients did not reveal any evidence of lymphopenia or neutrophilia. Moreover, an analysis of variance of the module expression levels did not detect any changes related to cell count variability (see below), further supporting our hypothesis that the observed biological themes reflected functional changes in cell states, rather than leukocyte redistribution or changes in blood composition.

### Host response correlated with infection severity

**Fig. 5:**
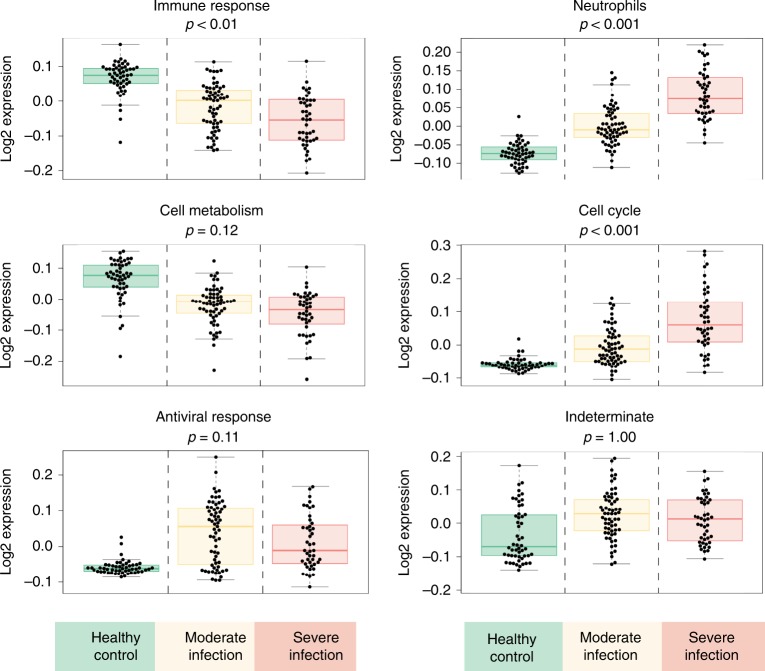
Analysis of variance of expression levels (eigengene) of six main modules. Boxplots of eigengene values of six main modules. *p* Values indicate statistically significant differences between moderate and severe influenza (Bonferroni correction for multiple testing). *Y*-axis shows normalized log_2_ expression levels. Box center line: median, box limits: upper and lower quartiles, whiskers: 1.5× interquartile range. The source data are available in Gene Expression Omnibus (GSE 101702)

Next, to identify the main determinants of module’s gene expression, we performed an analysis of variance (ANOVA) using the overall expression level of each module (“eigengene”) as dependent variable and host factors (infection severity, age, and cell counts) as independent variables. This analysis showed that infection severity, but not age or cell counts, was the main determinant of modular expression in neutrophils, cell cycle, and immune response modules (Fig. 5). As infection severity increased from moderate to severe, these three modules showed statistically significant changes in their gene-expression levels. In contrast, cell metabolism, antiviral response, and indeterminate modules did not show statistically significant changes in modular expression (cell metabolism *p* = 0.12, antiviral response *p* = 0.11, indeterminate *p* = 1.00), indicating that these modules were not associated with the severe infection. Put together, these findings suggest that all six modules played a role in the onset of influenza infection (from healthy state transitioning to moderate infection), but only neutrophils, cell cycle, and immune response modules were associated with severe, life-threatening illness.

To further quantify the extent of each module’s association with severe disease, we calculated the differences in the module’s expression levels (“eigengene differentials”) between healthy state and moderate influenza, or between moderate influenza and severe influenza (Fig. 3c). This calculation revealed that, as infection severity increased from moderate to severe, the neutrophils module displayed the highest increase in modular expression. Since moderate and severe influenza patients did not differ in their absolute neutrophil counts (Supplementary Fig. 1), this increase likely reflected differences in neutrophil activation, rather than changes in neutrophil cell numbers.

### Neutrophil module showed neutrophil activation and migration

Further examination of the neutrophils module confirmed the presence of neutrophil activation, as evidenced by the detection of a neutrophil transcriptomic signature that is typically found in activated neutrophils homing toward infected lung tissue in acute lung injury^24^. This “activation” signature included an increased expression in genes involved in delayed apoptosis (*BCL2A1* and *SMPD3*), activated inflammatory pathways (*CLEC4D*, *GPR84*, *S100A12*, and *S100A8*) and increased neutrophil extracellular traps (*GADD45A*, *HIST1H4H*, and *HIST2H2AA4*) (Fig. 4a).

Activated neutrophils usually migrate toward the infected lung after influenza infection^25^. Consistent with this, we detected genes within the neutrophils module that are involved in chemotaxis, neutrophil–endothelium interaction and transendothelial migration. These migration genes, include *CD177* (neutrophil adhesion to endothelium and transmigration), *PVRL2* (transendothelial migration), *VNN1* (hematopoietic cell trafficking), *GPR84* (neutrophil chemotaxis), *MMP9* (neutrophil activation and migration), *S100A8* and *S100A12* (neutrophil recruitment, chemotaxis and migration) (Fig. 4a).

Since an increased neutrophil activation is sometimes associated with a cytokine-mediated inflammation in influenza infection^26^, we searched for transcriptomic evidence of “hypercytokinaemia”, a phenomenon frequently reported in severe influenza patients^14^. We did not find strong evidence of hypercytokinaemia on a gene-expression level (Fig. 4d). The absence of pro-inflammatory gene upregulation might be related to the sampling method used in this study, in which only peripheral blood was measured (whereas the infected lung is thought to be the main source of inflammatory cytokines).

### Immune response module revealed broad downregulation

In contrast to the neutrophils module, the immune response module showed a broad downregulation in gene expression (Figs. 4b and 5). This downregulation included key genes involved in innate and adaptive immunity, such as *GZMH*, *KLRB1*, *SH2D1B* (NK cells function), CCR7 (T cells activation/homing), *TCF7* (T cell differentiation), *IL2RB* (T cell-mediated immunity), *CX3CR1* (lymphocyte migration and monocytes survival), *GNLY* (T cell cytotoxicity), *GZMB* (cytotoxic lymphocyte-induced apoptosis of infected cells), *KLRG1* (NK cells-mediated activation of virus-specific CD8 lymphocytes), and *FGFBP2* (cytotoxic lymphocyte-mediated immunity).

Downregulation of gene expression was also observed in genes associated with CD4/CD8 T cells, which are critical cells required for viral clearance. This downregulation was more marked in the severe group than the moderate group (Supplementary Fig. 3). In addition, a number of genes related to T cell receptor signaling were also downregulated (Supplementary Fig. 4).

The above findings indicated reduced expression across both innate and adaptive immunity, a phenomenon typically observed in patients with severe infection^27^. *HLADR* downregulation, a well-established marker of immune downregulation^28^, showed that this downregulation was more marked in the severe group than the moderate group (Supplementary Fig. 5). This finding was also consistent with the ANOVA analysis above (Fig. 5) and was confirmed by pathway analysis (see below). It was also in keeping with our observation that severe influenza patients had a significantly higher incidence of secondary complications known to be associated with immune suppression, including bacterial co-infection and multiple organ failure ^29^ (Table 1).

### Cell cycle pathway suggested host–virus interaction

**Fig. 6:**
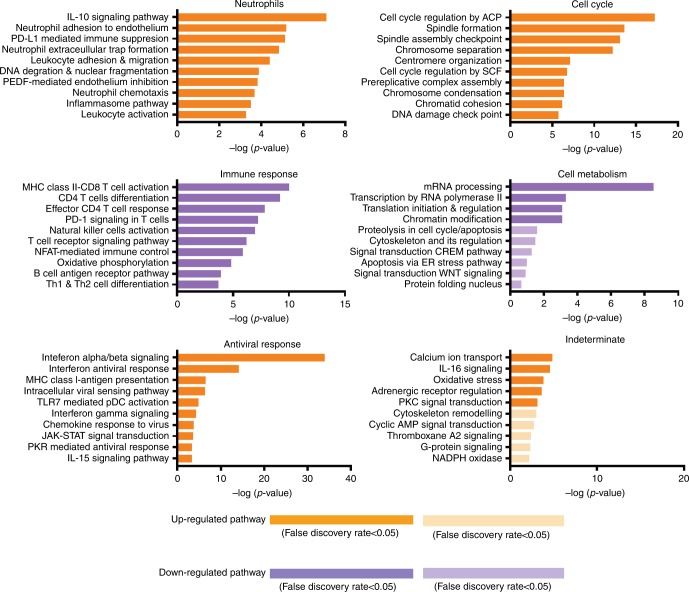
Pathway analysis. Top ten pathways ranked by *p* values (horizontal bars) in six main modules. Horizontal axis denotes statistical significance as measured by minus logarithm of *p* values. False discovery rate greater than 0.05 is indicated by faded horizontal bars. Detailed statistics of the pathway analysis are also given in Supplementary Fig. 6. The source data are available in Gene Expression Omnibus (GSE 101702)

The cell cycle module displayed the second highest increase in modular expression from moderate-to-severe influenza. Examination of the genes within this module revealed changes in several critical transition points in cell cycle pathways (Fig. 6), which were previously identified in influenza infection^30^^–32^. These changes, included delayed transition of host cell from G0 to G1 phase and downregulation of Cycling D, a key regulator of early cell cycle G1 phase^31,32^. These changes are in keeping with findings of several previous studies, which showed that cell cycle transition points could be manipulated by influenza virus to facilitate viral survival and to escape host inhibition^30–32^.

### Pathway analysis confirmed module biological themes

Pathway analysis was performed in all 6 modules, including the neutrophils (783 genes), cell cycle (408 genes), immune response (2759 genes), cell metabolism (645 genes), antiviral response (522 genes), and indeterminate (325 genes) modules. This analysis confirmed our earlier findings in that the neutrophils module showed neutrophil migration toward injury (e.g., chemotaxis, neutrophil–endothelium interaction, and transendothelial migration), the immune response module showed downregulated immune pathways, the cell cycle module, and the antiviral module showed upregulated pathways (Fig. 6, Supplementary Fig. 6).

The pathway analysis revealed a marked concordance between our observations and influenza-related mechanisms previously reported, including changes in a large number of interferon-derived genes (Figs. 4c and 5). In addition to the interferon pathways, the pathway analysis identified other pathways that have been previously reported, including increased IL-10 signaling^33^, reduced innate immunity^7^, impaired CD4 and CD8 response^4–6^ and reduced natural killer cell function^8^. The pathway analysis also identified several mechanisms that have not been previously reported, including downregulation of several key T cell functions (e.g., cell activation/differentiation and Th1/Th2 differentiation) and reduced antigen presentation (MHC class II), both of which were consistent with our earlier findings of downregulated immune function.

### Internal and external validation

We performed leave-one-out cross-validation of three main modules (neutrophils, cell cycle, and immune response) (Supplementary Method). To be consistent with the original analysis, all samples included in the WGCNA (63 moderate influenza and 44 severe influenza samples) were also used in the cross-validation. The correct classification rate (i.e., predicting whether an unidentified sample belongs to a moderate or severe patient) was computed during the cross-validation. To ensure the findings were not affected by the choice of the prediction algorithm, we deployed seven machine-learning algorithms during the cross-validation process (Supplementary Table 1). The results of this cross-validation showed that all three modules performed well in predicting class membership (moderate vs. severe). Notably, most algorithms consistently identified the neutrophils module as the best performing module in distinguishing between moderate influenza and severe influenza samples (Supplementary Table 1).

**Fig. 7:**
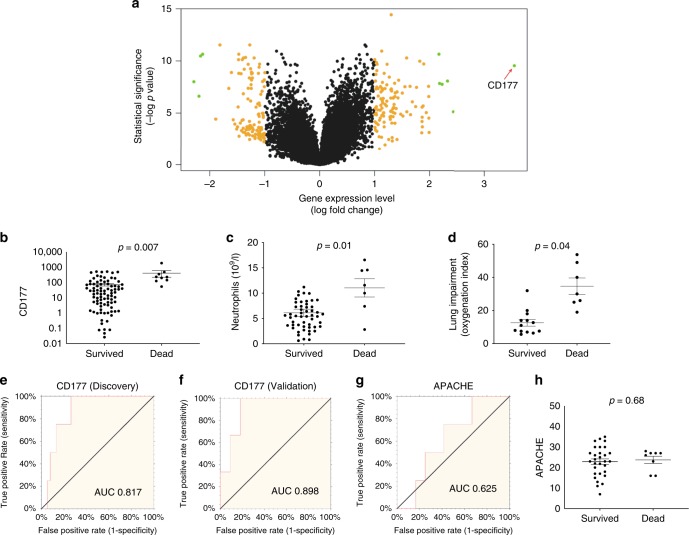
Neutrophil activity predicts patient outcomes. **a** *CD177* is the most highly differentially expressed gene in severe infection. Volcano plot from microarray analysis (the discovery set) comparing moderate infection (*n* = 63) and severe infection (*n* = 44); differentially expressed genes are colored. The source data is available in Gene Expression Omnibus (GSE 101702). **b** *CD177* gene expression levels in survivors and nonsurvivors. *CD177* gene-expression levels (measured by quantitative real-time PCR in peripheral blood) between survivors and nonsurvivors. **c** Neutrophil counts in survivors and nonsurvivors. Neutrophil counts in the peripheral blood of severe influenza patients. **d** Lung impairment in survivors and nonsurvivors. The degree of lung impairment is indicated by the oxygenation index using established method (calculated as inspired oxygen concentration in atmospheric air multiplied by mean airway pressure, divided by partial pressure of oxygen in arterial blood)^45^. A higher oxygenation index indicates more severe lung impairment. **e**–**h** Mortality prediction using *CD177* expression levels or APACHE scores. Receiver–operator characteristics (ROC) curves of *CD177* gene-expression (measured by quantitative real-time PCR) to predict mortality in **e** the discovery set (95% confidence interval: 0.602–0.921) and **f** the validation set (95% confidence interval: 0.769–0.957). In **g**, **h**, APACHE scores are also shown (ROC 95% confidence interval: 0.230–0.843). APACHE is a clinical mortality risk prediction score composing of age, premorbid comorbidities and acute physiological derangements. **b**–**d**, **h** *p* Values were calculated by Mann–Whitney *U* test. Error bars represent means and standard errors. Source data for **b**–**h** are provided as a Source Data Excel file

We performed RT-PCR validation of the microarray findings on a small number of selected genes (see Supplementary Method). The RT-PCR measurements showed that, on these selected genes, microarray data correlated well with PCR data (Supplementary Fig. 8). We also assessed the protein expression of the *CD177* gene, the most upregulated differentially expressed gene in patients with severe influenza (Fig. 7a). Blood samples from influenza infected patients in a separate study (*n* = 26) were used for this purpose. Using flow cytometry analysis (Supplementary Method), we found that CD177 protein expression in blood also distinguished between moderate and severe influenza (*p* = 0.038) (Supplementary Fig. 9).

We performed external validation by downloading the full microarray dataset of a similar study (GSE 111368). This external dataset was comparable to our study in terms of sample size (107 vs. 109) and infection severity (severe, moderate, and healthy controls). Because the two datasets used different microarray platforms (Agilent vs. Illumina), we restricted our analysis to the probes represented in both microarray platforms. This analysis revealed three major findings. Firstly, among the 20 most highly upregulated, differentially expressed genes (moderate vs. severe), there was 80% concordance between the two datasets (Supplementary Table 2). Notably, many of these concordant genes encode proteins relating to neutrophil functions, indicating there was biological similarity between the two datasets among the top-ranking genes. Secondly, among all the differentially expressed genes common to both datasets (a total of 356 upregulated and downregulated genes), pathway analysis revealed two major themes similar to our original finding—for the upregulated genes, the main biological theme in both datasets was excessive neutrophil activation, and for the downregulated genes, the main biological theme in both datasets was a reduced immune response (Supplementary Table 3). Thirdly, representative genes related to these two themes (neutrophils and the immune response) discriminated between moderate and severe influenza in both datasets (Supplementary Fig. 10).

### Neutrophil activity predicted patient outcome

The findings presented heretofore indicate that, among the six main modules, the neutrophils module showed the strongest association with disease severity (Figs. 3c and 5). Recent animal studies suggest several possible mechanisms to explain this strong association, including the possible role of neutrophils extracellular traps (NETs) in causing lung injury in influenza^25,34^. In keeping with this, we found that the top 30 differentially expressed genes (Supplementary Fig. 7) contained many NETs-related genes, including defensin, myeloperoxidase, elastase, neutrophil gelatinase and cathepsin, all of which are well-known markers of NETs ^35^.

Given the prominent roles displayed by neutrophil-related genes in our data, and the previously reported etiological link between neutrophil activity and influenza severity^25,34^, we hypothesized that changes in neutrophil state could predict patient outcome. To test this hypothesis, we performed a separate study to evaluate whether changes in neutrophil gene expression could accurately predict influenza mortality in our patients. *CD177* was selected as a representative gene of neutrophil activation state because *CD177* is a neutrophil-specific marker ^36^ and importantly, it was the most highly differentially expressed gene in severe infection (Fig. 7a). The association between *CD177* expression levels (measured by real-time quantitative PCR) and patient outcomes (e.g., mortality) was measured by area-under-the receiver–operator curve, using the established method^37^. Two independent datasets were used in this analysis; a “discovery set” consisted of 41 patients from the original microarray analysis (the other remaining samples did not contain enough RNA), and a “validation set” consisted of 47 patients for whom blood samples were not used in the original analysis (Fig. 1, Supplementary Table 4).

This analysis showed that the *CD177* gene had a significantly higher expression in nonsurvivors than survivors (Fig. 7b). Furthermore, nonsurvivors had a significantly higher number of neutrophils in their peripheral blood (Fig. 7c), and this was associated with a greater degree of oxygenation failure in their lung (Fig. 7d). Importantly, area under the curve (AUC) analysis of the receiver operating characteristic curve (Fig. 7e, f) showed that *CD177* expression predicted influenza-related deaths in both the discovery set (AUC 0.817, 95% confidence interval: 0.602–0.921) and the validation set (AUC 0.898, 95% confidence interval: 0.769–0.957). Based on Youden index, CD177 displayed sensitivity 0.80 (95% confidence interval 0.28–0.99), specificity 0.75 (95% confidence interval 0.58–0.88), positive predictive value 0.31 (95% confidence interval: 0.18–0.48), and negative predictive value 0.96 (95% confidence interval 0.82–0.99). In addition, *CD177* expression better discriminated survivors vs. nonsurvivors than the traditional clinical score APACHE (AUC 0.625, 95% confidence interval: 0.230–0.843) (Fig. 7g, h). Collectively, these findings support our hypothesis that an excessive neutrophil activation state was linked to a poor outcome in influenza infection.

## Discussion

Numerous host factors contribute to the pathogenesis of influenza infection. However, the relative contribution of individual host factor remains poorly defined^9^. This uncertainty represents a major roadblock in the design of host-based drug therapy, which has long been recognized as a priority in the global effort to reduce influenza-related mortality. Here, we assembled a large cohort of influenza-patients with well characterized phenotypes in whom we identified six modules of the systemic host response associated with influenza infection. Three of the six modules (neutrophils, cell cycle and immune response) showed an association with disease severity. Of these three modules, the neutrophils module displayed the highest correlation with disease activity. Within the neutrophils module, we identified several key pathogenic pathways in circulating neutrophils, including neutrophil extracellular traps formation and neutrophil-inflammation, both of which have been previously implicated in the pulmonary pathogenesis of severe influenza^25,34^. Furthermore, we found that excessive neutrophil activity was present in patients with severe respiratory failure and that increased expression of neutrophil activation marker could predict influenza-related fatality. Collectively, these findings provide a clinically relevant framework to better understand influenza pathogenesis and revealed a previously under-appreciated link between neutrophils and severe influenza infection.

An important caveat is warranted in the interpretation of our findings—that causality cannot be inferred from our findings. This is due to two main reasons. First, this is a single time point study; no longitudinal data is provided. Second, the patients were recruited relatively late in the course of their illness; patients from an early phase of the infection were under-represented in the cohort (since symptoms were not severe enough or the patient had not sought medical attention). Due to these reasons, it remains impossible to conclude whether the observed findings (e.g., neutrophil activation) were a cause or a consequence of severe disease. A longitudinal analysis could address these issues since samples can be collected at symptom onset and continuously until respiratory failure occurs (from subclinical state, to symptom development and finally to respiratory failure). This approach captures the time-dependent dynamics of the host response, which may be more informative.

The study findings highlight the dichotomous roles of host response and add nuance to our current understanding of influenza infection. Immunologists have long agreed that the same host factors responsible for lung injury are also critical for efficient control of influenza virus replication^2^. For example, excessive neutrophil response is associated with severe influenza, as observed in our study and in previous reports^25^. However, a robust neutrophil response is also a necessary part of the host defense^35^. Indeed, this study shows that patients with moderate influenza had evidence of neutrophil activation, albeit only modestly. This finding consolidates the current view that the host response operates on a continuum (from benefit to harm), and that preserving some degree of a beneficial host response along this continuum should be an important consideration in the design of host-directed therapy.

The main strengths of this study are its large sample size, its relevant clinical context and the global view of the host response generated from within this context. The large sample size enabled us to examine the full spectrum of infection severity, ranging from moderate influenza to severe influenza-induced lung injury. The use of whole blood sampling helped us generate an integrated view of the leukocyte-mediated host response by preserving the interactions between different leukocyte subsets, which provides a realistic measure of how immune cells interact during infection. The transcriptome analysis provided a broad coverage of gene functions, thereby allowing a systematic interrogation of many pathways (although we are cognizant of the limitations of transcriptomic analysis, including the need for further validation on a protein level). Collectively, these study design features allowed us measure influenza host response in a clinically relevant context across a broad array of immune cells.

The findings of this study are in line with previous animal studies, which suggested that neutrophils play a prominent role in causing severe disease in influenza infection^25,34^. However, neutrophils are unlikely to be the only host factors that influence disease severity; a more plausible scenario is that severe disease results from the interplay between neutrophils and other host factors^38^. In this study, we attempted to capture this interplay by using an “unsupervised” network analysis approach (WGCNA). Typically, an “unsupervised” approach does not use a priori phenotype information (e.g., infection severity) or prior assumption about the underlying biology^39^. Thus, it allowed the generation of an unbiased view of all pathways expressed in circulating leukocytes. Another advantage of the WGCNA approach is that it treats the host response as a “whole system”, rather than individual components with few interconnectivities. With these methodological considerations, an integrated, global view of host factors was generated from our analysis allowing us to identify the main host factors associated with disease severity.

This global analysis allowed us to identify neutrophils as the dominant host factors associated with patient outcome. Similar findings have been provided by studies conducted in animal models^25,34^. It remains an intriguing question to ask whether modulation of these host factors, such as the neutrophil response, could modify disease outcome. This question has recently been investigated in an animal model^40^; but a randomized controlled clinical trial in humans would provide the most definitive answer.

As with all observational studies, this study could be biased due to additional confounders. Patient level confounders were likely; however, in our analysis, both the moderate and severe groups were well balanced in baseline characteristics. Cell level confounders were also possible; however, our analyses had carefully considered and allowed for the possible bias introduced by cell number variability. As a further step to minimize bias, we included total leukocyte counts and neutrophil counts in the ANOVA model, which showed that cell counts had no effect on gene-expression levels. Another potential limitation of this study was that pulmonary leukocytes were not measured in our patients; this measurement would have expanded the insights gained. However, it is worthwhile pointing out that performing bronchoscopy in these patients (in order to retrieve pulmonary leukocytes in infected lung) was difficult to justify on ethical grounds because these patients were critically ill and were at a high risk of rapid decompensation during bronchoscopy.

In conclusion, this study helps identify a previously under-appreciated link between neutrophils and severe disease in influenza infection. Further mechanistic study is needed to unravel the mechanism underlying the neutrophil-related host response and thus help identify novel therapeutic target.

## Methods

### Study design and participants

We recruited patients from 20 hospitals (Australia, Canada and Germany) during the period from 2009 to 2016 (Fig. 1). The eligibility criteria, included (1) age greater than 18 years and (2) World Health Organization definition of influenza-like illness (fever of 38 °C or higher, cough and illness onset within the last 10 days). All eligible patients were assessed by an admitting physician for likelihood of influenza infection. Patients with a high likelihood of infection, based on history and clinical features, were enrolled into the study. Airway samples (nasopharyngeal swab, throat sample or sputum) and a 2.5 ml peripheral blood sample (into PAXgene tubes) were obtained from each study participant. Routine investigations were performed, as determined by the admitting physician. Airway samples were tested for bacterial pathogens and common respiratory viruses. Blood samples in PAXgene tubes were later processed for microarray analysis (Agilent 8× 60k Human V3). The study was approved by the institutional review board of each participating institution. Informed consent was obtained from all participants.

### Microbiological and virology testing

Nasopharyngeal, sputum, urine, and blood samples were obtained at admission. In patients admitted to intensive care unit, additional respiratory samples were obtained from bronchoalveolar lavage or tracheal aspirates. Standard microbiological testing was performed in these samples, including sputum Gram stain and culture. Testing for atypical respiratory pathogens (*Chlamydophila pneumoniae*, *Mycoplasma pneumoniae*, and *Legionella pneumophila*) was performed in selected patients at the discretion of treating physicians. All patients were tested for respiratory viruses using nucleic acid PCR. The PCR panel included primers for influenza A, influenza B, respiratory syncytial virus, rhinovirus, parainfluenza virus, and human metapneumovirus. Only patients who were positive for influenza virus were included in the main analysis.

### Case definitions

Influenza infection was defined as the identification of either influenza A (H1N1 or H3N2) or influenza B using nucleic acid amplification (real-time PCR) in a participant with clinical features consistent with influenza infection. Moderate influenza infection was defined as disease with significant symptoms resulting in presentation to an emergency department, but without the need for invasive respiratory support. Severe influenza infection was defined as influenza infection with significant respiratory failure requiring endotracheal intubation and mechanical ventilation. Case assignment (moderate or severe group) was done retrospectively, based on a full review of clinical features, virology testing and laboratory investigations, and was performed by two consultant physicians independent of those physicians admitting the participants. Discrepancies in phenotype assignments were resolved by a third physician.

### RNA extraction, normalization, and microarray analysis

In each sample, whole-blood RNA was extracted from PAXgene tubes as per manufacturer’s protocol (QIAGEN PreAnalytiX—Blood RNA version 2; 2015). After checking RNA integrity on Bioanalyzer (Agilent Technologies; Waldbronn, Germany), 100 ng of total RNA were applied for Cy3-labeling reaction using the one color Quick Amp Labeling protocol (Agilent Technologies; Waldbronn, Germany). Labeled cRNA was hybridized to Agilent 8 × 60k Human V3 (Design ID: 072363) microarrays for 16 h at 68 °C and scanned using the Agilent DNA Microarray Scanner. Microarray data were then analyzed using the R software package (version 3.1.3). Preprocessing steps included background correction, adding an offset of 50, quantile normalization and annotation using the limma package and Agi4x44PreProcess packages. Multigroup comparisons and identification of differentially expressed probe sets were performed using limma with Benjamini and Hochberg correction for multiple testing. Differentially expressed genes were identified based on an adjusted *p* value of <0.05, and exhibiting more than a twofold difference in expression levels ([log^2^] > 1). Full dataset of the gene-expression data is available at the National Centre for Biotechnology Information Gene Expression Omnibus (GEO accession number GSE101702).

### Weighted gene co-expression network analysis

*Overview of weighted gene co-expression network analysis (WCGNA*)—to identify groups of co-regulated genes (“modules”) associated with infection severity, we applied unsupervised hierarchical clustering across microarray data of all samples (Fig. 1). WGCNA was used as a clustering method. The main advantage of WGCNA is that it takes into account the inter-relationship between and within networks, thereby allowing a more biologically meaningful interpretation^41^. WGCNA organizes the transcriptome into tightly correlated sets of transcripts. It first generates a pairwise similarity matrix based on expression correlation, which is transformed into an “adjancy matrix”. This results in a highly modular network topology. Hierarchical clustering (using average linkage) is then used to define modules within this network. The overall expression of a given module is summarized by the “eigengene” (the first principle component of the gene-expression matrix). We used the eigengene values to calculate the “eigengene differentials” (the difference in eigengene values between illness severity), which quantifies each module’s association with severe disease. ANOVA was used to determine the association between the eigengene (dependent variable) and infection severity, age, gender, and cell counts (independent variables). Modules that were significantly associated with disease severity were further investigated by pathway analysis (see below).

*Procedure of WCGNA*—we used the R package WGCNA (version 1.5.1) and we selected the top 10,000 most variant probesets as input and applied a soft threshold of 10 for network construction. This allows the construction of Pearson’s correlation matrices for all pairs of genes. The correlation matrices were then transformed into an adjacency matrix using a power function *f*(*x*) = *x*^*β*^. The parameter *β* of the power function was determined such that the resulting adjacency matrix was scale free. The adjacency matrix was transformed into a topology overlap, which measures the connectivity among all the genes in the network. Based on this topology overlap, hierarchical average linkage clustering was then used to construct the dendrogram and identify gene co-expression modules that contain the maximal sets of inter-connected genes. More detailed information regarding the WCGNA procedure can be found in WCGNA website: (https://labs.genetics.ucla.edu/horvath/CoexpressionNetwork/). In this study, the following parameters were used for WCGNA analysis: TOMType: unsigned network, minModuleSize: 30, reassignThreshold: 0.25, power: 10 and reassignThreshold: 0.

### Pathway analysis

We identified the biological theme in each module by performing a pathway analysis using the MetaCore (https://portal.genego.com/). MetaCore is a web-based, integrated software suite for functional analysis of omics data. It uses a topology approach (similar to WCGNA), which takes into account the interaction between and within pathways, the position of the genes within each pathway and the interaction between genes. We uploaded genes from each module (identified earlier in WCGNA) into MetaCore and used the pathway enrichment analysis option within the software. The enrichment analysis identifies coordinated changes in gene-expression in a predefined group of functionally related pathway genes, based on a curated database of known protein–protein interaction, signaling and metabolic pathways. To ensure our results were biologically meaningful, pathway analysis was restricted to modules which contained at least 300 genes.

### Digital cell quantification

We inferred the immune cell quantities in each blood sample using the decomposition-based digital cell quantification (DCQ) method, which has been previously validated in influenza infection^4^. DCQ uses a large reference library (“immune cell compendium”) consisting of the transcriptional profiles of a predefined set of genes that encode cell surface markers (i.e., that identify immune cell types by fluorescence-activated cell sorting). Here, we used the DMAP expression data for the immune cell compendium and the R package ComICS for DCQ analysis (https://cran.r-project.org/web/packages/ComICS/index.html). The DCQ output was visualized as a heatmap (Supplementary Fig. 2), which provides a quantitative measure of the “relative” abundance of each immune cell types, namely the change in cell quantity between a sample of a given phenotype (e.g., severe influenza) and a sample in steady state (e.g., healthy control).

### Sample size calculation

The sample size was chosen to have 90% power in detecting differentially expressed genes between severity groups (moderate vs. severe), assuming a false-discovery rate of 0.05. Based on gene-expression variance and effect size estimated from our previous studies^42,43^, we calculated that a sample size of 46 in each severity group would be required. In total, 750 individuals with flu-like illness were needed to obtain 46 influenza-positive subjects in each severity group (moderate or severe), based on the estimation that (1) only 25% of flu-like illness individuals would be test-positive for influenza virus (estimated from our pilot data), and (2) among those positive for influenza, 13% would develop moderate or severe influenza infection (based on publicly available data of 70,000 laboratory confirmed hospitalized patients with influenza infection^44^).

### Statistical analysis

Comparisons between two severity groups (moderate vs. severe) were calculated using the unpaired two-tailed Student’s *t* test or the nonparametric Mann–Whitney *U* test where appropriate. To assess outcome prediction in the independent validation study, area-under-curve (and its confidence interval) was calculated using the receiver–operator-characteristic curve analysis, as implemented in NCSS software (2018, Utah, USA, ncss.com/software/ncss). General statistical analyses were performed using PRISM and R software package, and bioinformatic analyses were performed using R software package.

### Reporting summary

Further information on research design is available in the Nature Research Reporting Summary linked to this article.

## Supplementary information

Supplementary Information

Reporting Summary

Source Data

## Data Availability

Microarray data underlying Figs. 2, 3, 4, 5, 6, 7a and Supplementary Figs. 2, 3, 4, 5, 6, 7, 10, 11 are available at Gene Expression Omnibus (Accession numbers: GSE 101702 and GSE 111368); [https://www.ncbi.nlm.nih.gov/geo/]. Source Data underlying Fig. 7b–h and Supplementary Figs. 1, 8 are provided as Source Data file. All other data are available from the corresponding author upon reasonable requests.

## Code availability

Codes and derived data used for the analysis of the microarray expression (normalized data matrix, derived data, PCA, WGCNA, heatmaps, volcano plots, DCQ analysis, boxplots for gene expression, scatter plots, boxplots for module eigengenes, VENN diagrams for modules) is publicly available at [10.22000/152].
