# Supplementary material for: Neutrophils-related host factors associated with severe disease and fatality in patients with influenza infection
Source: Nat Commun. 2019 Jul 31;10:3422. doi: 10.1038/s41467-019-11249-y (PMC6668409; doi:10.1038/s41467-019-11249-y)
Supplement: Supplementary file 1 — Supplementary Information [file 41467_2019_11249_MOESM1_ESM.pdf]

Supplementary Information

Neutrophils-related host factors associated with severe disease and fatality in patients with influenza infection

Tang et al.

SUPPLEMENTARY FIGURES

Supplementary Figure 1 – Total leukocyte and neutrophil counts

Supplementary Figure 2 – Digital cell quantification (DCQ)

Supplementary Figure 3 – CD4 and CD8 gene expression

Supplementary Figure 4 – T cell subsets receptor signalling

Supplementary Figure 5 – HLADR expression

Supplementary Figure 6 – Summary statistics of pathway analyses

Supplementary Figure 7 – Top 30 differentially expressed genes

Supplementary Figure 8 - Correlation between qPCR and microarray

Supplementary Figure 9 – CD177 protein expression

Supplementary Figure 10 – External validation

Supplementary Figure 11 – Overlap between modules

Supplementary Figure 1

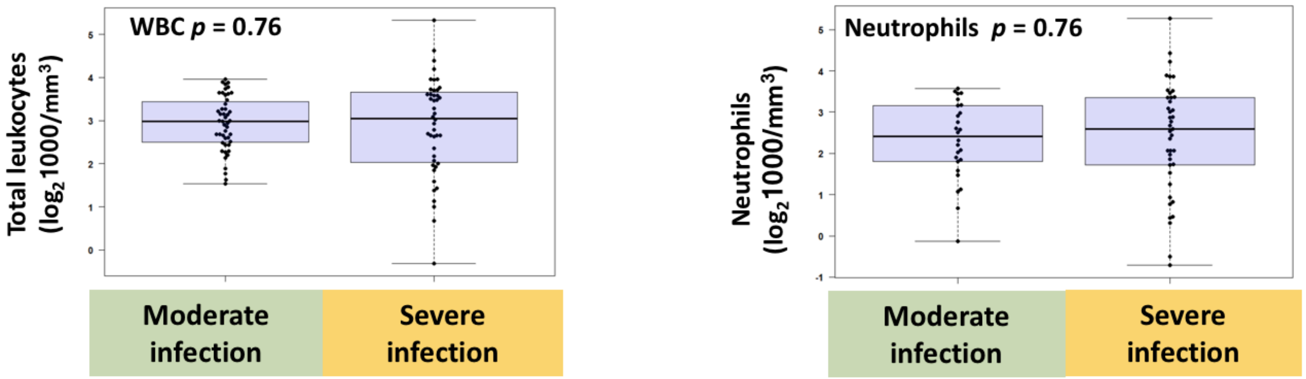

Supplementary Figure 1. White blood cell counts and **neutrophil cell counts** are not statistically different between moderate and severe infection groups. Box center line: median, box limits: upper and lower quartiles, whiskers: 1.5x interquartile range. *p* values are calculated by Mann-Whitney U test. Source data are provided in source files: Source File Supp Fig 1.txt and File Instruction Supp Fig 1.pdf.

Supplementary Figure 2

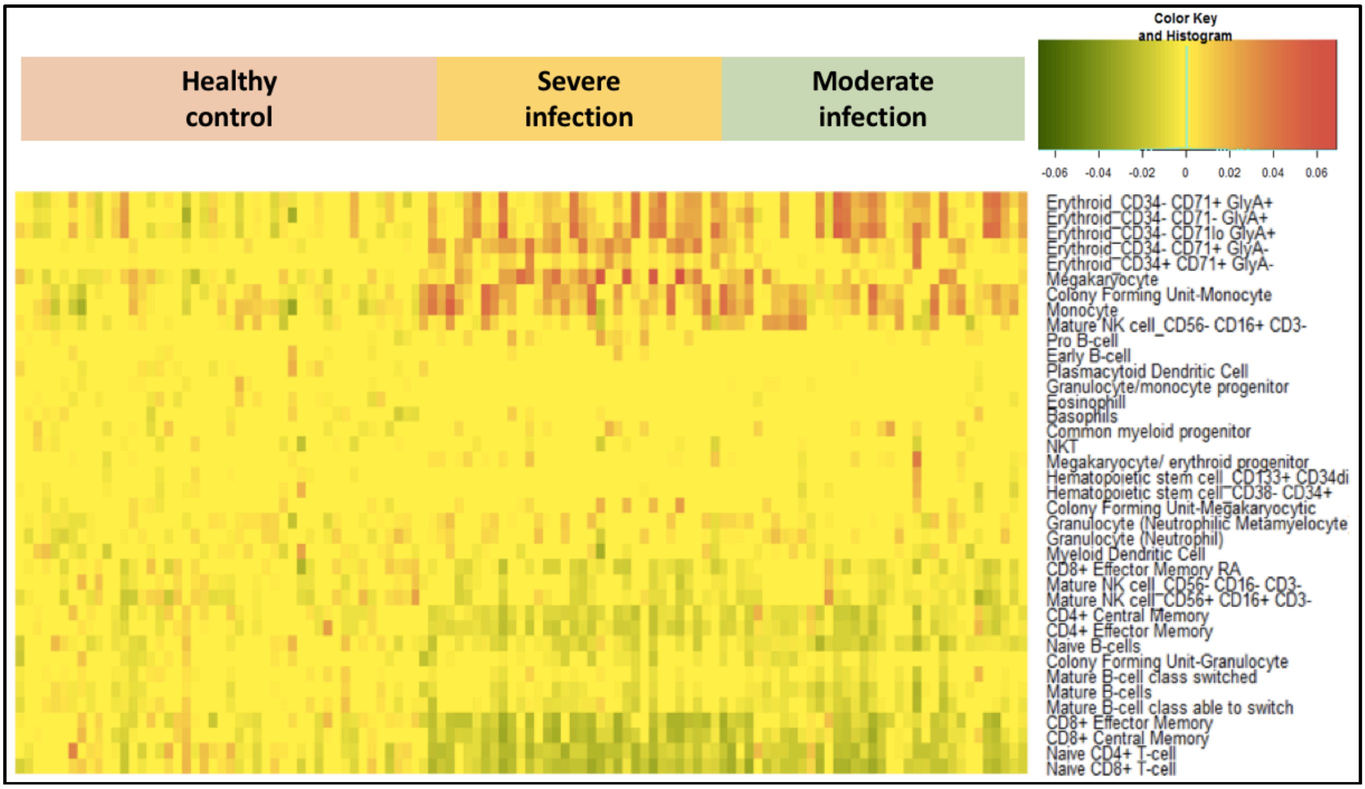

Supplementary Figure 2. Relative abundance of immune cell populations, as determined by Digital Cell Quantification (DCQ) from gene expression values using the DMAP dataset as reference for leukocyte gene-expression signatures (details are outlined in **Method**). No obvious difference, except for pro-erythroblasts, was observed between moderate and severe groups for most leukocyte subsets. The source data is available in Gene Expression Omnibus (GSE 101702).

Supplementary Figure 3

CD8A gene expression (p = 0.046)

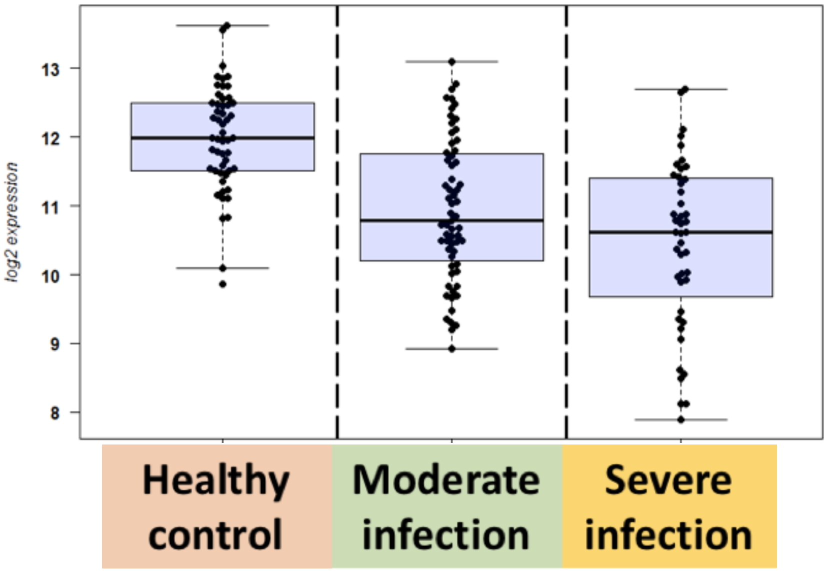

CD4 gene expression (p < 0.01)

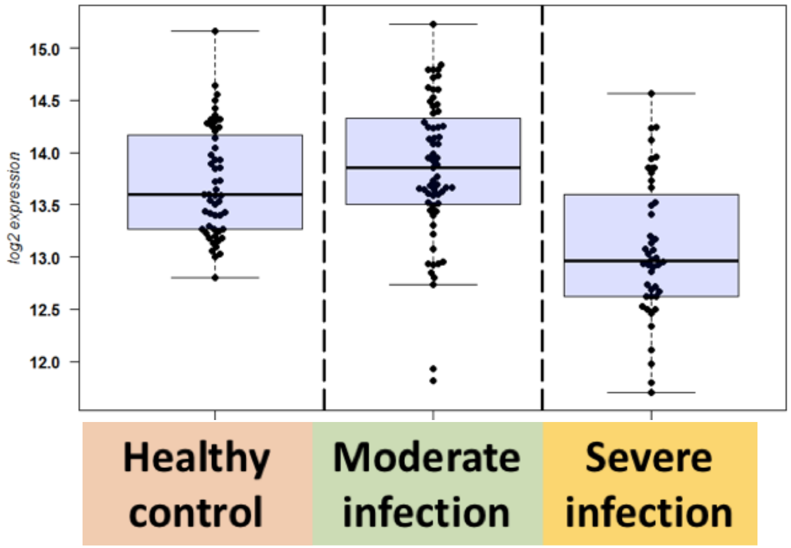

Supplementary Figure 3. Boxplots of CD4 and CD8A gene-expression. p-values indicate statistically significant differences between moderate and severe influenza (adjusted for multiple testing by Bonferroni method). Y-axis shows normalized $\log_2$ expression levels. Box center line: median, box limits: upper and lower quartiles, whiskers: 1.5x interquartile range. The source data is available in Gene Expression Omnibus (GSE 101702).

Supplementary Figure 4

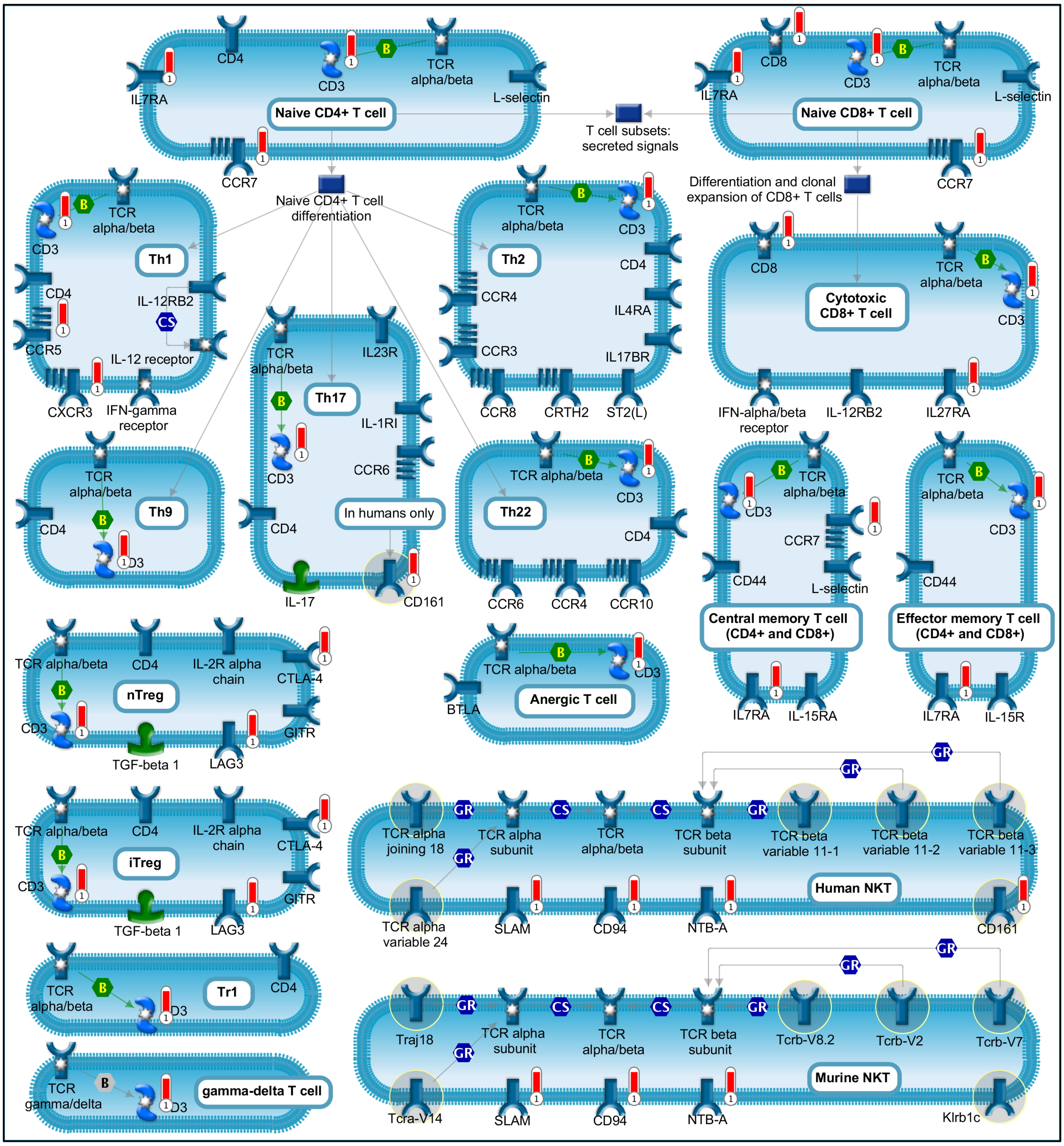

Supplementary Figure 4: **T-cell subsets with genes encoding for cell surface molecules for receptor signalling.** Only statistically significant genes (down-regulated) are shown; they are highlighted by red vertical columns. The source data is available in Gene Expression Omnibus (GSE 101702).

Supplementary Figure 5

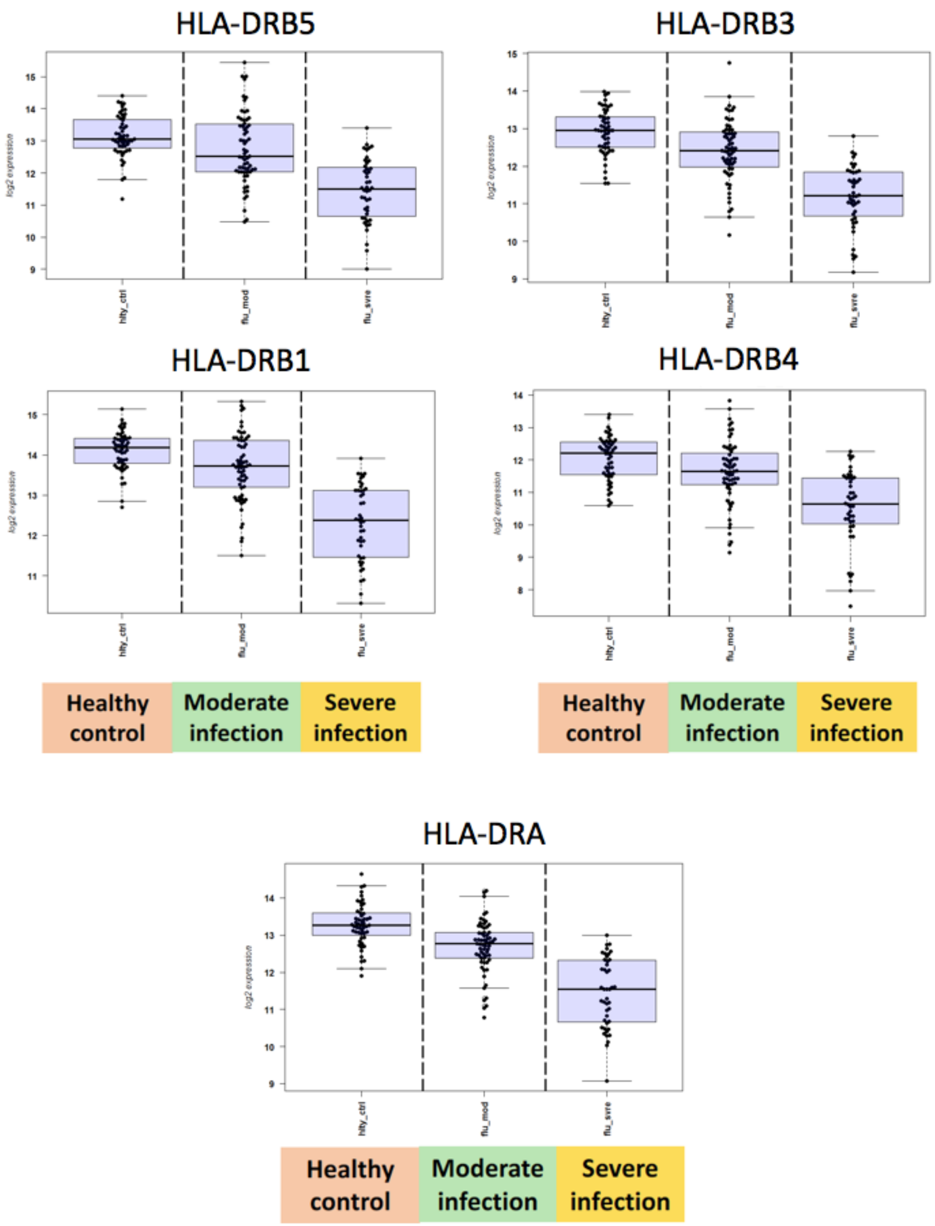

Supplementary Figure 5: **HLADR genes** (expression levels from microarray data) were significantly more down-regulated in the severe group compared to the moderate group ($p<0.001$, adjusted for multiple testing by Bonferroni method). The downregulation of HLA-DRA gene, the most well-established clinical biomarker of immune suppression, was confirmed by qPCR measurement in patients' peripheral blood samples. Box center line: median, box limits: upper and lower quartiles, whiskers: 1.5x interquartile range. The source data is available in Gene Expression Omnibus (GSE 101702).

Supplementary Figure 6

Pathways	Gene enrichment in pathways	p values	log p values	False discvoery rate	Modules
IL-10 signaling pathway	12/62	8.08E-08	7.093	7.73E-05	Neutrophils
Neutrophil adherence to endothelium	11/77	6.56E-06	5.183	1.81E-03	
PD-L1 mediated immune suppression	10/64	7.64E-06	5.117	1.81E-03	
Neutrophil extracellular trap formation	7/31	1.51E-05	4.827	2.68E-05	
Leukocyte adhesion & migration	10/77	4.06E-05	4.392	5.75E-03	
DNA degradation & nuclear fragmentation	6/30	1.35E-04	3.870	1.38E-02	
PEDF-mediated endothelium inhibition	6/31	1.64E-04	3.786	1.46E-02	
Neutrophils chemotaxis	7/46	2.27E-04	3.644	1.81E-02	
Inflammasome pathway	6/35	3.29E-04	3.483	2.36E-02	
Leukocyte activation	7/53	5.55E-04	3.255	3.43E-02	
Cell cycle regulation by ACP	14/32	6.71E-18	17.168	2.31E-15	Cell cycle
Spindle formation	12/33	2.52E-14	13.594	4.33E-12	
Spindle assembly checkpoint	12/36	8.54E-14	13.064	9.79E-12	
Chromosome separation	11/32	6.51E-13	12.182	5.60E-11	
Centromere organization	7/26	8.20E-08	7.083	5.64E-06	
Cell cycle regulation by SCF	7/29	1.87E-07	6.724	1.07E-05	
Prereplicative complex assembly	7/32	3.89E-07	6.407	1.91E-05	
Chromosome condensation	6/21	4.91E-07	6.363	2.11E-05	
Chromatid cohesion	6/22	6.67E-07	6.173	2.55E-05	
DNA damage check point	6/26	1.96E-06	5.708	6.12E-05	
MHC class II-CD8 T cell activation	30/101	1.03E-10	9.987	4.34E-08	Immune response
CD4 T cell differentiation	27/90	6.97E-10	9.168	1.96E-07	
Effector CD4 T cell response	20/60	1.55E-08	7.810	3.26E-06	
PD-1 signalling in T cells	18/53	5.86E-08	7.232	9.86E-06	
Natural killer cells activation	18/55	1.12E-07	6.950	1.57E-05	
T cell receptor signaling pathway	17/55	6.38E-07	6.202	7.67E-05	
NFAT-mediated immune control	16/51	1.09E-06	5.839	1.15E-04	
Oxidative phosphorylation	22/103	1.46E-05	4.837	9.43E-04	
B cell antigen receptor pathway	21/110	1.28E-04	3.892	4.91E-03	
Th1 & Th2 cell differentiation	11/40	2.00E-04	3.700	6.01E-03	
mRNA processing	21/160	3.11E-09	8.515	4.47E-07	Cell metabolism
Transcription by RNA polymerase II	13/159	5.21E-04	3.287	3.12E-02	
Translation initiation	11/127	8.68E-04	3.065	3.12E-02	
Chromatin modification	11/127	8.68E-04	3.065	3.12E-02	
Proteolysis in cell cycle/apoptosis	8/126	2.56E-02	1.593	3.08E-01	
Cytoskeleton and its regulation	6/85	3.23E-02	1.492	3.58E-01	
Signal transduction CREM pathway	6/98	5.79E-02	1.239	5.21E-01	
Apoptosis via ER stress pathway	5/89	1.06E-01	0.977	6.55E-01	
Signal transduction WNT signaling	8/177	1.27E-01	0.897	6.55E-01	
Protein folding nucleus	3/58	2.24E-01	0.650	9.21E-01	
Inteferon alpha/beta signaling	29/64	1.34E-34	33.873	7.17E-32	Antiviral response
Inteferon antiviral response	15/52	7.81E-15	14.107	2.09E-12	
MHC class I-antigen presentation	9/54	3.88E-07	6.411	4.63E-05	
Intracellular viral sensing pathway	7/28	4.32E-07	6.365	4.63E-05	
TLR7 mediated pDC activation	9/84	1.70E-05	4.770	1.52E-03	
Interferon gamma signaling	7/56	5.56E-05	4.255	3.73E-03	
Chemokine response to virus	8/92	2.23E-04	3.652	1.12E-02	
JAK-STAT signal transduction	5/32	2.29E-04	3.639	1.12E-02	
PKR mediated antiviral response	6/57	4.98E-04	3.303	2.05E-02	
IL-15 signaling pathway	4/22	5.51E-04	3.259	2.11E-02	
Calcium ion transport	5/48	1.72E-05	4.846	5.93E-03	Indeterminate
IL-16 signaling	5/55	3.37E-05	4.554	5.93E-03	
Oxidative stress	4/42	1.81E-04	3.807	2.12E-04	
Adrenergic receptor regulation	4/47	2.81E-04	3.615	2.47E-02	
PKC signal transduction	4/65	9.74E-04	3.106	4.90E-02	
Cytoskeleton remodelling	3/31	1.18E-03	2.975	5.21E-02	
cAMP signal transduction	3/38	2.15E-03	2.715	6.87E-02	
Thromboxane A2 signaling	3/50	4.71E-03	2.373	1.03E-01	
G-protein signaling	2/17	5.82E-03	2.267	1.14E-01	
NADPH oxidase	3/59	7.48E-03	2.171	1.15E-01	

Supplementary Figure 6. Summary statistics of pathway analyses; the fraction in the second column "Gene enrichment in pathways" denote the number of genes identified in each pathway (numerator) and the total number of known genes in the pathway (denominator). The source data is available in Gene Expression Omnibus (GSE 101702).

Supplementary Figure 7

EntrezGeneID	GeneSymbol	GeneName	adj.P.Val	NETs	Neutrophil	Function
57126	CD177	CD177 molecule	2.99E-10			Neutrophil transmigration
10562	OLFM4	olfactomedin 4	7.33E-06			Chemotaxis and cell adhesion
56729	RETN	resistin	8.41E-09			Chemotaxis and cell adhesion
3240	HP	haptoglobin	1.73E-08			
56729	RETN	resistin	1.44E-08			Chemotaxis and cell adhesion
199675	MCEMP1	mast cell-expressed membrane protein 1	2.24E-11			
1669	DEFA4	defensin, alpha 4, corticostatin	0.000714951			Neutrophil granules peptide/protein
1991	ELANE	elastase, neutrophil expressed	3.02E-05			Neutrophil granules protease
4353	MPO	myeloperoxidase	2.55E-07			Neutrophil granules peptide/protein
3250	HPR	haptoglobin-related protein	2.71E-08			
3934	LCN2	lipocalin 2	9.03E-06			Neutrophil granules peptide/protein
6283	S100A12	S100 calcium binding protein A12	1.93E-10			Neutrophil granules peptide/protein
383	ARG1	arginase 1	2.06E-06			
4057	LTF	lactotransferrin	0.000205743			Neutrophil granules peptide/protein
1668	DEFA3	defensin, alpha 3, neutrophil-specific	2.53E-05			Neutrophil granules peptide/protein
383	ARG1	arginase 1	2.64E-06			
1088	CEACAM8	carcinoembryonic antigen-related cell adhesion molecule 8	6.50E-05			Chemotaxis
1511	CTSG	cathepsin G	0.000772606			Neutrophil granules peptide/protein
6283	S100A12	S100 calcium binding protein A12	1.04E-10			Neutrophil granules peptide/protein
8876	VNN1	vanin 1	3.49E-08			
671	BPI	bactericidal/permeability-increasing protein	2.41E-06			
4318	MMP9	matrix metallopeptidase 9 (gelatinase B, 92kDa gelatinase, 92kDa t	8.32E-07			Neutrophil granules peptide/protein
7850	IL1R2	interleukin 1 receptor, type II	1.42E-05			
79623	GALNT14	polypeptide N-acetylgalactosaminyltransferase 14	2.71E-08			
6590	SLPI	secretory leukocyte peptidase inhibitor	7.47E-06			
122402	TDRD9	tudor domain containing 9	5.15E-11			
762	CA4	carbonic anhydrase IV	2.95E-08			
6286	S100P	S100 calcium binding protein P	1.89E-05			Neutrophil granules peptide/protein
306	ANXA3	annexin A3	1.21E-07			
8993	PGLYRP1	peptidoglycan recognition protein 1	1.26E-05			

Supplementary Figure 7: **Top 30 differentially expressed genes** (ranked by expression levels) between moderate and severe infections. *p*-values were corrected for multiple testing by Benjamin/Hochberg method ("adj.P.Val"). Neutrophil-related genes are coloured in red and NETs-related genes are coloured in pink. "NETs" denotes neutrophil extracellular traps. The source data is available in Gene Expression Omnibus (GSE 101702).

Supplementary Figure 8

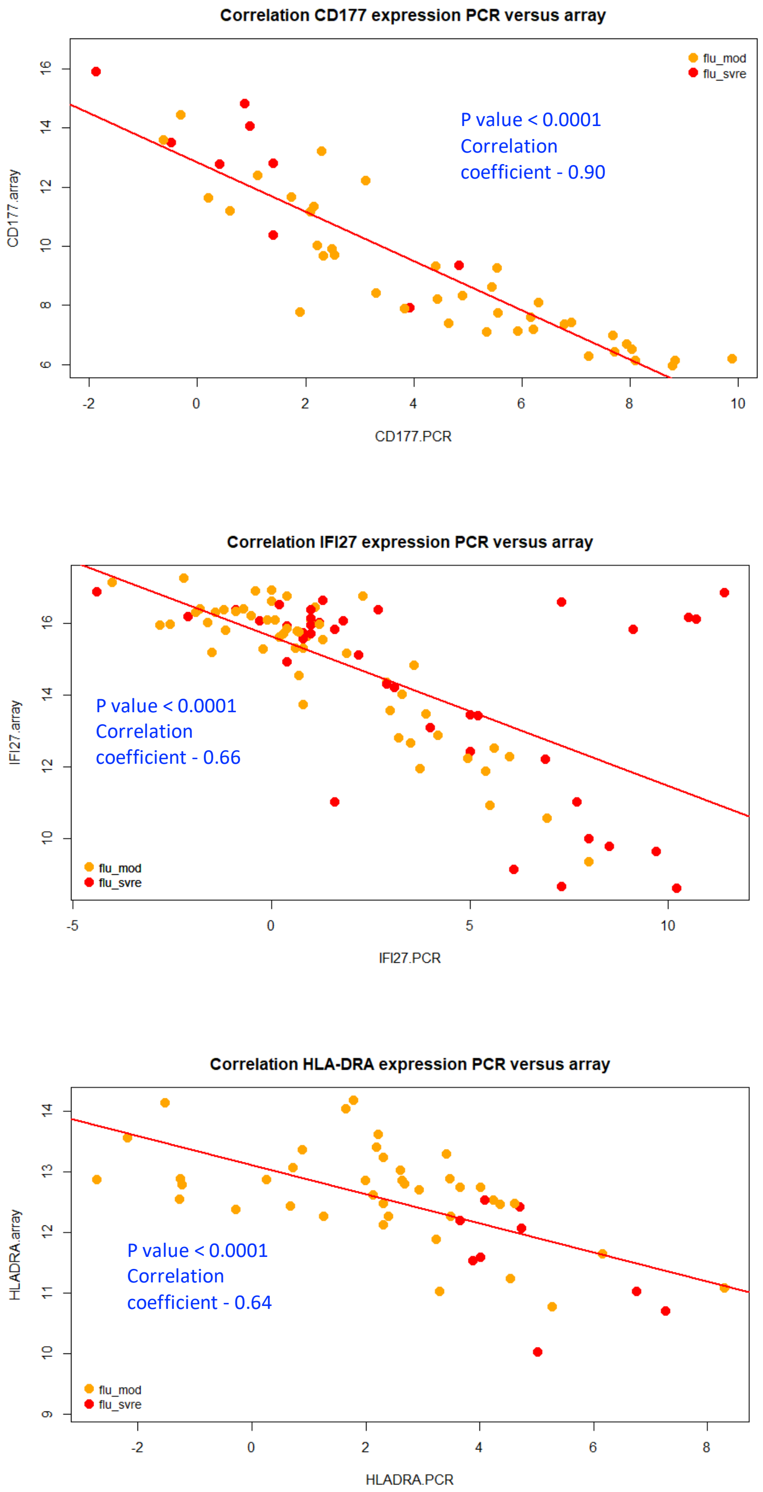

Supplementary Figure 8: **Gene expression levels correlated well between microarray and PCR**. Three representative genes (*CD177, IFI27, HLA*-DRA) were selected (see **Supplementary Method**). Y-axis: log_2 normalized gene-expression levels from the microarray data, X-axis: minus delta ct values from qRT-PCR analysis. “Moderate” denotes samples of moderate influenza patients and “Severe” denotes samples of severe influenza patients. Source data are provided in source files: Source File Supp Fig 8.txt and File Instruction Supp Fig 8.pdf.

Supplementary Figure 9

9a

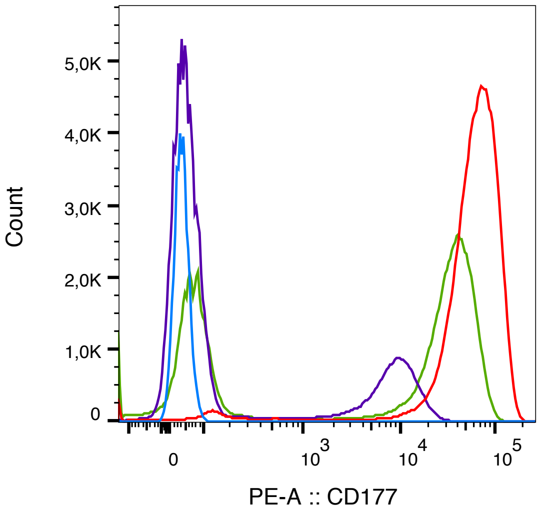

9b

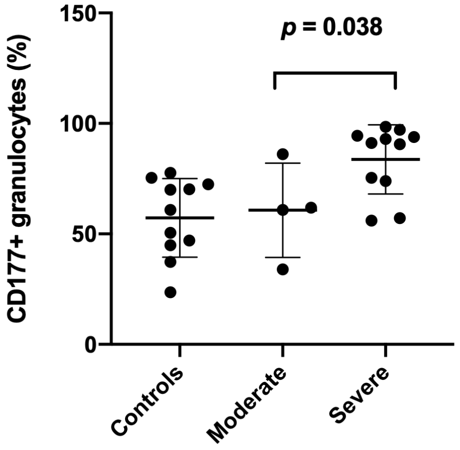

9c

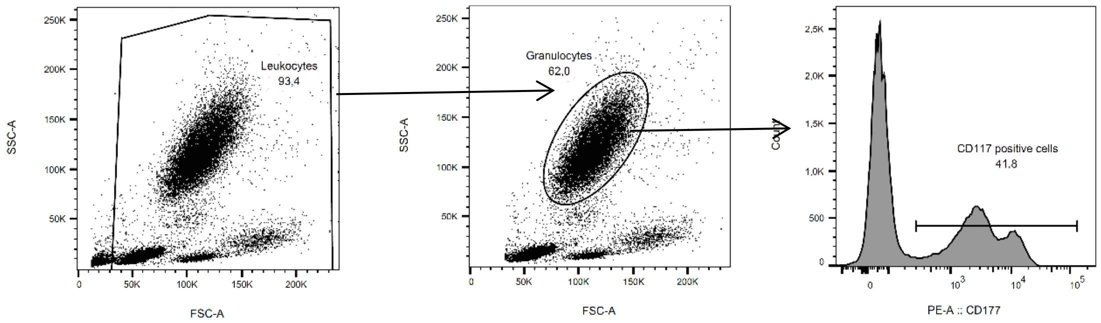

Supplementary Figure 9: **CD177 protein expression levels in circulating granulocytes**.

9a: Representative flow cytometry results of CD177 surface expression on granulocytes in healthy donor (violet colour) and in patient with moderate influenza infection (green colour) and severe influenza infection (red colour). Whole blood samples were incubated with anti-CD177 antibody (violet, green, red) or isotype control (blue colour) for 30 min (4°C), followed by red blood cells lysis and washing with PBS. Granulocytes were gated using SSC/FSC dot plot, then proportion of CD177+ granulocytes were evaluated using standard negative (left peak, CD177- cells) / positive (right peak, CD177+ cells) gating strategy.

9b: Percentage of CD177+ granulocytes were analysed using FlowJo with healthy volunteers (n=11), moderate influenza infection (n=4) and severe influenza infection (n=11). *p* value was calculated using upaired *t* test.

9c: Percentage of CD117 positive granulocytes was determined using followed gating strategy: SSC/FCS – cell debris exclusion (leukocytes selection); SSC/FCS (from previous gated population without debris) for granulocytes selection, histogram for final evaluation of CD177 expression (positive cells gating strategy).

Source data for Supplementary Figure 9b are provided as a Source Data Excel file.

Supplementary Figure 10

Current dataset **External dataset**

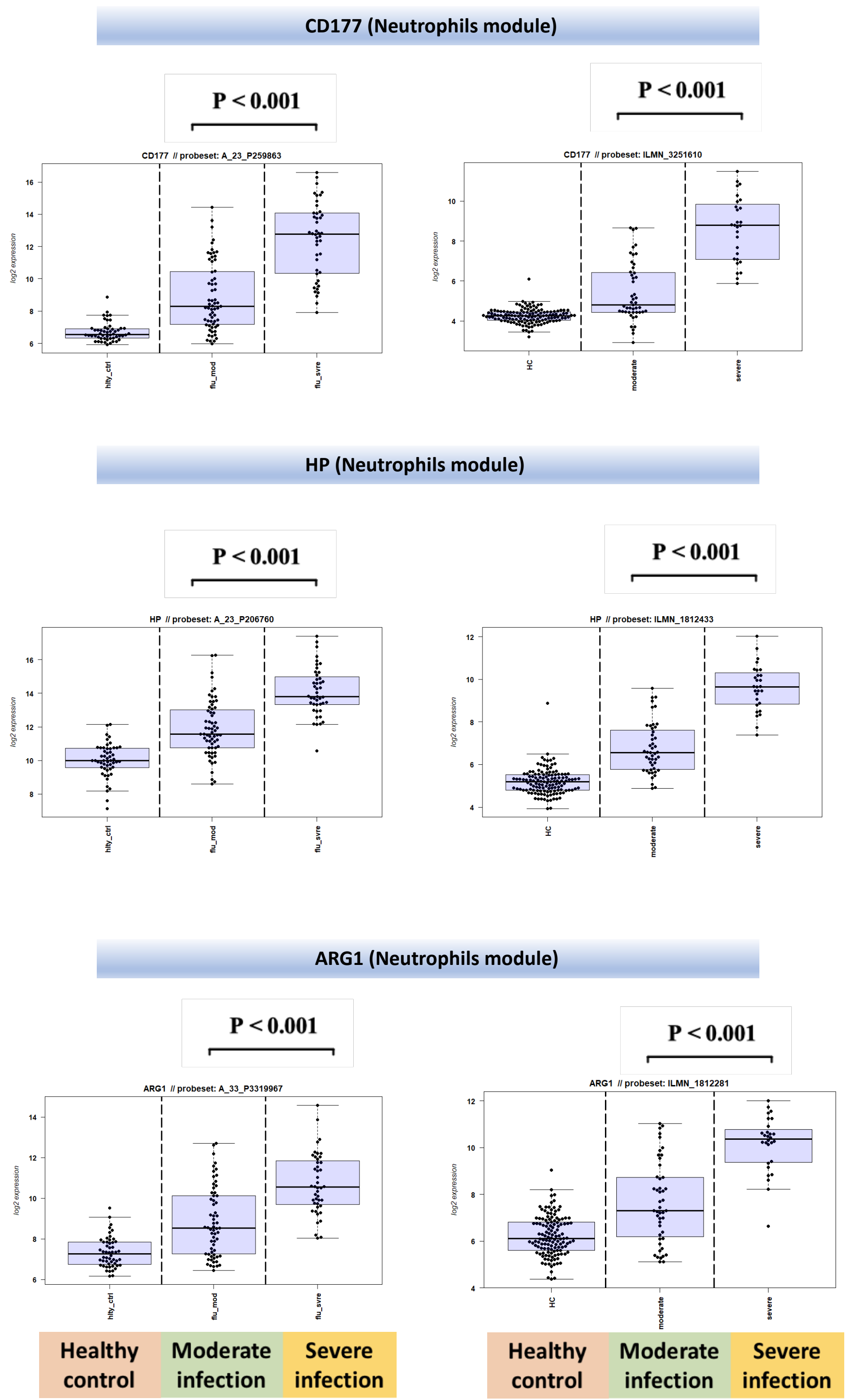

Supplementary Figure 10: External validation – expression profiles of neutrophil-derived genes (*CD177, HP,* ARG1) distinguish between moderate and severe infection in both datasets. *p* values were calculated by comparing between the moderate and the severe infection groups, and have been adjusted for multiple testing by Bonferroni method. Box center line: median, box limits: upper and lower quartiles, whiskers: 1.5x interquartile range. Source data: current dataset GSE101702; external dataset GSE111368, both available in GEO gene expression database (https://www.ncbi.nlm.nih.gov/geo).

Supplementary Figure 10 (continue)

Current dataset **External dataset**

S100P (Immune response module)

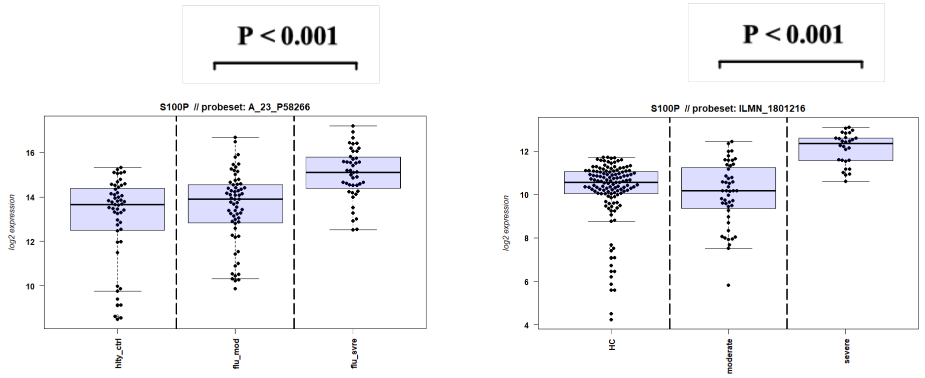

HLA-DRA (Immune response module)

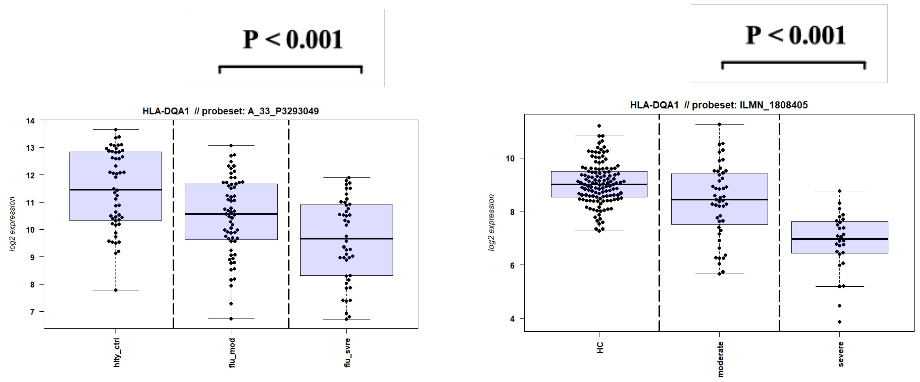

SREBF1 (Immune response module)

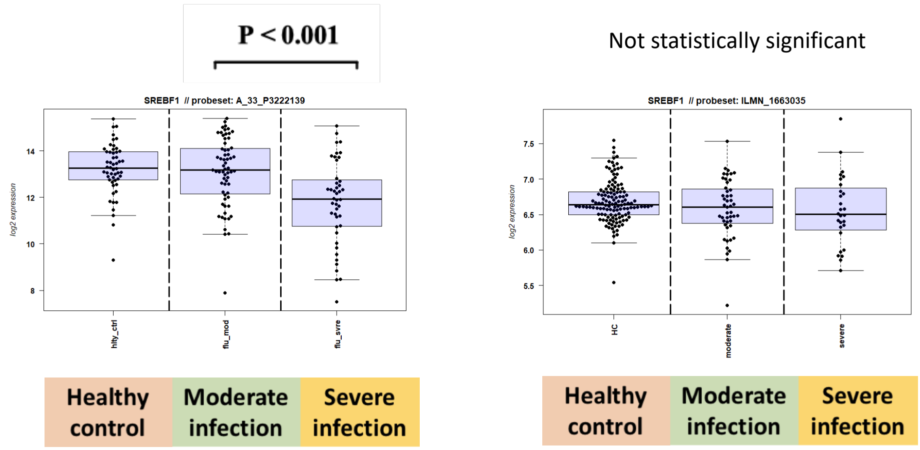

Supplementary Figure10 (continue): External validation – expression profiles of immune response-derived genes *S100P, HLA-DRA*, but not SREBF1, distinguish between moderate and severe infection in both datasets.

Supplementary Figure 11

Module overlap on a probe level

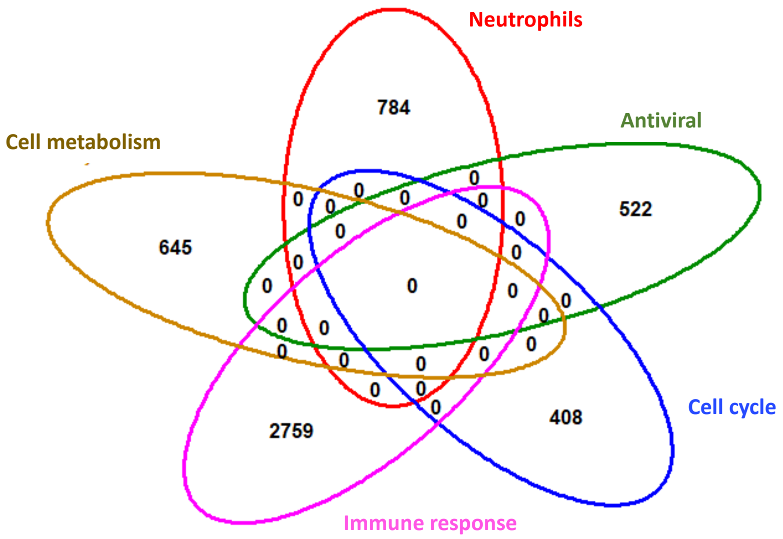

Module overlap on a gene level

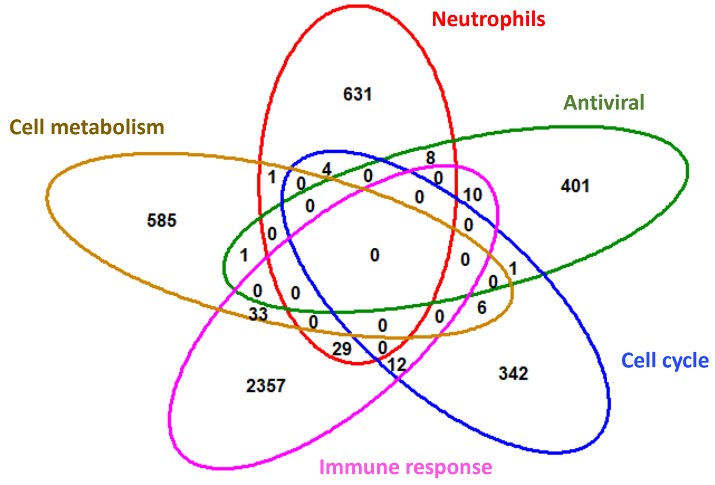

Supplementary Figure 11: **Venn diagrams** – overlap between modules on a probe level **(top)** and on a gene level **(bottom)**. The source data is available in Gene Expression Omnibus (GSE 101702).

SUPPLEMENTARY TABLES

Supplementary Table S1: Cross-validation - results

Supplementary Table S2: External validation – top genes

Supplementary Table S3: External validation – top pathways

Supplementary Table S4: Validation cohort characteristics

Supplementary Table S1: Cross-validation to distinguish moderate/severe influenza cases.

	Compound Covariate Predictor	Diagonal Linear Discriminant Analysis	1-Nearest Neighbour	3-Nearest Neighbour	Nearest Centroid	Support Vector Machine	Bayesian Compound Covariate Predictor
Performance in leave-one-out cross-validation (correct classification rate)							
Neutrophils	0.80	0.80	0.76	0.80	0.79	0.83	0.90
Cell cycle	0.77	0.76	0.79	0.83	0.77	0.87	0.88
Immune response	0.69	0.69	0.79	0.81	0.68	0.87	0.90
Statistical significance (based on 1000 random permutations)							
Neutrophils	P < 0.01	P < 0.01	P < 0.01	P < 0.01	P < 0.01	P < 0.01	P < 0.01
Cell cycle	P < 0.01	P < 0.01	P < 0.01	P < 0.01	P < 0.01	P < 0.01	P < 0.01
Immune response	P < 0.01	P < 0.01	P < 0.01	P < 0.01	P < 0.01	P < 0.01	P < 0.01
Area-under-curve (AUC)							
Neutrophils	0.89	0.88	*	*	*	*	0.88
Cell cycle	0.82	0.83	*	*	*	*	0.81
Immune response	0.82	0.82	*	*	*	*	0.82
Specificity							
Neutrophils	0.83	0.83	0.83	0.84	0.83	0.87	0.62
Cell cycle	0.81	0.81	0.89	0.90	0.81	0.92	0.44
Immune response	0.68	0.68	0.87	0.91	0.68	0.91	0.49
Sensitivity							
Neutrophils	0.77	0.77	0.66	0.75	0.75	0.77	0.48
Cell cycle	0.71	0.68	0.66	0.73	0.71	0.79	0.39
Immune response	0.71	0.71	0.66	0.68	0.68	0.82	0.48
Positive predictive value							
Neutrophils	0.76	0.76	0.73	0.77	0.75	0.81	0.47
Cell cycle	0.72	0.71	0.81	0.84	0.72	0.88	0.33
Immune response	0.61	0.61	0.78	0.83	0.60	0.86	0.40
Negative predictive value							
Neutrophils	0.84	0.84	0.78	0.83	0.83	0.85	0.63
Cell cycle	0.80	0.79	0.79	0.83	0.80	0.87	0.51
Immune response	0.77	0.77	0.79	0.80	0.75	0.88	0.57

* Computation for AUC is not possible for several prediction methods (1-Nearest Neighbour, 3-Nearest Neighbour, Nearest Centroid and Support Vector Machine).

Supplementary Table S2. Top 20 most upregulated genes common to both datasets.

Current dataset (107 infected patients)	External dataset (109 infected patients)
**CD177**	**OLFM4**
**OLFM4**	**ELANE**
**RETN**	**LOC653600**
**HP**	**CD177**
MCEMP1	**MMP8**
**DEFA4**	**DEFA4**
**ELANE**	ZDHHC19
**MPO**	**RETN**
HPR	**MPO**
**LCN2**	**HP**
S100A12	**BPI**
**ARG1**	**CEACAM6**
**LTF**	**CTSG**
**DEFA3**	**CEACAM6**
**CEACAM8**	DEFA1B
**CTSG**	**LCN2**
VNN1	PGLYRP1
**BPI**	**LTF**
**MMP9**	**ARG1**
**IL1R2**	**IL1R2**

Note: genes that are common to both datasets are highlighted in **red**.

Supplementary Table S3. Top 10 pathways common to both datasets.

Pathways (ranked by statistical significance)	Gene enrichment in pathways*	*p* values	False discovery rate	Direction of gene expression changes
Transcriptional regulation of neutrophils development	9/32	1.127e-8	1.206e-5	Upregulated
Leukocyte activation and differentiation	10/50	6.033e-8	3.228e-5	Upregulated
MHC class II antigen presentation	9/41	1.196e-7	4.244e-5	Down-regulated
Neutrophil extracellular trap formation	8/31	1.587e-7	4.244e-5	Upregulated
G-CSF induced neutrophil differentiation	7/30	2.044e-6	4.373e-4	Upregulated
T lymphocyte cell receptor signalling pathway	9/58	2.676e-6	4.772e-4	Down-regulated
N-RAS regulated CD4 T-lymphocyte activation	7/33	4.067e-6	6.216e-4	Down-regulated
CD4 T-lymphocyte differentiation	8/49	6.572e-6	8.791e-4	Down-regulated
T lymphocyte cell subset activation	8/52	1.040e-5	1.236e-3	Down-regulated
Integrin dependent eosinophil activation	8/58	2.379e-5	2.545e-3	Upregulated

* The fraction in the column “Gene enrichment in pathways” denote the number of genes identified in each pathway (numerator) and the total number of known genes in the pathway (denominator).

Supplementary Table S4. Comparison between the discovery and validation cohorts.

	Discovery	Validation	*p* values*
**Number**	107	47	
**Gender** (males/females)	44/63	20/27	0.99
**Age/years** (mean + SD)	50 (18)	54.7 (21)	0.54
**Duration of onset**	4.8 days	4.9 days	0.11
**Symptoms**			
- Cough	62 (58%)	45 (96%)	<0.001*
- Fever/chills	48 (45%)	35 (74%)	<0.001*
- Dyspnoea	58 (54%)	36 (77%)	0.01*
- Malaise/aches	50 (47%)	43 (91%)	<0.001*
**Pre-existing illnesses**			
- Asthma	17 (16%)	11 (23%)	0.27
- Chronic lung disease	18 (17%)	13 (28%)	0.13
- Ischemic heart disease	18 (17%)	12 (26%)	0.27
- Diabetes	17 (16%)	5 (11%)	0.46
- Cancer/on chemotherapy	7 (6.5%)	1 (2.1%)	0.44
**Virology**			
- Positive influenza PCR	107 (100%)	47 (100%)	0.99
- Influenza A subtype	93 (87%)	43 (91.5%)	0.59
- Influenza B subtype	14 (13%)	4 (8.5%)	0.59
- antiviral treatment (Tamiflu)	43 (40%)	18 (38%)	0.86
**Respiratory support**			
- Mechanical ventilation	42 (39%)	6 (13%)	0.01*
- Non-invasive support (CPAP)	2 (1.9%)	1 (2.1%)	0.99
**Secondary complications**			
- Bacterial pneumonia	7 (6.5%)	1 (2%)	0.44
- Shock	12 (11%)	4 (8.5%)	0.78
- Acute renal failure	7 (6.5%)	1 (2%)	0.44
- Multiple organ failure	14 (13%)	4 (8.5%)	0.59
**Outcomes**			
- Hospitalization	89 (83%)	33 (70%)	0.09
- Admission to ICU	51 (48%)	6 (13%)	0.001*
- Length of ICU stay	18 days	15 days	0.79
- Length of hospital stay	14 days	9.4 days	0.97
- Death	9 (8.4%)	5 (11%)	0.76

* *p* values are calculated by comparing moderate and severe groups using Mann-Whitney test for continuous variables or Chi-square test for categorical variables. ICU denotes intensive care unit.

SUPPLEMENTARY METHODS, DISCUSSION AND REFERENCES

Supplementary Methods

- Cross-validation
- External validation
- RT-PCR
- Flow cytometry
- Coinfection patients

Supplementary Discussion

Supplementary References

SUPPLEMENTARY METHODS

Cross-validation

In the main text, three main modules (neutrophils, immune response and cell cycle) were identified by the weighted gene coexpression network analysis (WGCNA) as statistically significant in distinguishing between moderate and severe influenza (Figure 3e). To assess the robustness and stability of this finding, we performed an internal cross-validation on these three modules using the same set of samples used in the original WGCNA analysis (the "discovery cohort"). During this cross-validation, we evaluated, one module at a time, the "likely" prediction error to be expected when applying these modules in a different, independent dataset. We calculated the statistical significance of prediction accuracy by randomly assigning class membership to each sample (moderate or severe), and then computed the probability of obtaining the same finding as would be expected by chance. The results of these findings are presented in **Supplementary Table 1**.

"Leaving-one-out" method

We choose the "leave-one-out" method over other cross-validation methods (e.g. k-fold validation or bootstrap) because of the modest sample size of our cohort and the fact that the "leave-one-out" method is less computationally intensive than other methods. The "leave-one-out" method omits one sample at a time, and for each sample omitted, the entire analysis is repeated from scratch to select the best combination of predictive genes within the module. This process generates a gene-expression "classifier", which is a subset of genes from within the module that would yield the highest prediction accuracy. This process is repeated multiple times, withholding all of the samples (within the cohort) one at a time.

Calculating statistical significance

The *p*-values for the cross-validated misclassification error rate are provided in the **Supplementary Table 1**. To calculate the *p*-values, class labels (moderate vs. severe) are randomly assigned to each sample. For each random permutation of class labels, the entire cross-validation procedure is repeated to determine the cross-validated misclassification rate obtained by the classifier. The final *p*-value is the proportion of the random permutations that give as small a cross-validated misclassification rate as is observed with the real class labels. A total of 1000 permutations were performed to compute the *p*-values for the cross-validated misclassification rate.

Performance of cross-validation

In **Supplementary Table 1**, a number of parameters are provided to enable the readers to evaluate the cross-validation process. These parameters include correct classification rate, *p*-values, sensitivity, specificity, positive predictive value and negative predictive value. Area-under-the-receiver-operator-characteristics-curve (AUC) are also provided where possible (note: AUC calculation was not feasible for some algorithms).

Implementation of cross-validation

The cross-validation process was implemented by BRB ArrayTool (version 4.6). In the BRB ArrayTool, seven machine learning algorithms are deployed during the cross-validation

process. A brief description of each algorithm is provided in the table below. More details description can be found in the BRB ArrayTool manual (version 4.6).

Machine learning algorithms	Brief description
Compound covariate predictor	A modified weight voting method with each gene weighted by how well it discriminates between the classes (e.g. moderate vs. severe).
Diagonal linear discriminant analysis	Similar to the Compound Covariate Predictor above, but it ignores correlations among the genes in order to avoid over-fitting the data.
1-Nearest Neighbour	It searches for gene-expression similarity between a "training sample" and the nearest unidentified "prediction sample" in the dataset.
3-Nearest Neighbour	Similar to the above but instead of comparing one, it compares three "training samples" to the nearest unidentified samples in the dataset.
Nearest Centroid	It compares the gene-expression profile of an unidentified sample to the hub profile of all the identified samples (the "Centroid").
Support vector machine	It calculates a linear function of gene-expression intensities that best separates the class membership between different samples.
Bayesian Compound Covariate Predictor	It uses a Bayesian approach to calculate the posterior probability of an unidentified sample belonging to a known or identified class label.

External validation

To perform external validation, we downloaded the full microarray dataset of another study (GSE 111368). This study included influenza patients similar to our study and it used the same sampling approach (whole blood) as our study. However, potential bias may still arise due to differences in microarray platform, study design and different timing of sample collection between the two studies. To minimize this bias, we carefully controlled for aforementioned factors by restricting the analyse on parameters that are common to both studies, as follows;

(1). The two studies used different microarray platforms (Agilent vs. Illumina), we thus restricted the analyses to transcripts that are present in both microarray platforms.
(2). The external dataset (GSE 111368) included three time points in sample collection (admission, 2 days post admission and 4 weeks later). In our study, sample collection occurred on a single time point (admission). Hence, the analysis was restricted to one time point that is common to both studies (e.g. admission).
(3). The external dataset (GSE 111368) included three severity levels; "severity 1, 2 and 3". "Severity 1" corresponds to the "moderate influenza" group in our dataset, whereas "severity 3" corresponds to the "severe influenza" group in our study. Therefore, we analysed both studies on a like-for-like basis ("severity 1" and "severity 3" vs. "moderate influenza' and "severe influenza").

Analyses steps:
(1). We quantified the concordance in gene-expression profiles, between the two datasets, among the top most upregulated, differentially expressed genes.
(2). We search for similarity in biological themes between two datasets, as measured by pathway analysis of differentially expressed genes common to both datasets.
(3). We assessed whether signature genes could reliably discriminate between infection severity (moderate vs. severe) in the external dataset (GSE 111368).

For analysis (1), we calculated the extent of concordance by calculating the proportion of commonly shared genes between the two datasets (**Supplementary Table 2**). For analysis (2), a total of 365 differentially expressed genes (both upregulated and downregulated genes) were shared between the datasets (see Venn diagram below). We performed pathway analysis on these 365 genes and compared the biological themes of the pathway analysis (**Supplementary Table 3**) to the original analysis.

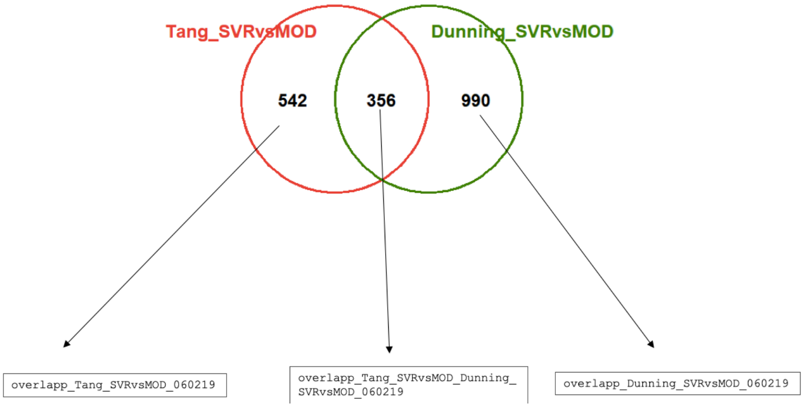

For analysis (3), we selected three representative modules from the original analysis, namely, neutrophils, immune response and antiviral modules. For each module, we identified the top most variant genes, and restricted our selection to only genes that were present in both microarray platforms (see diagram below).

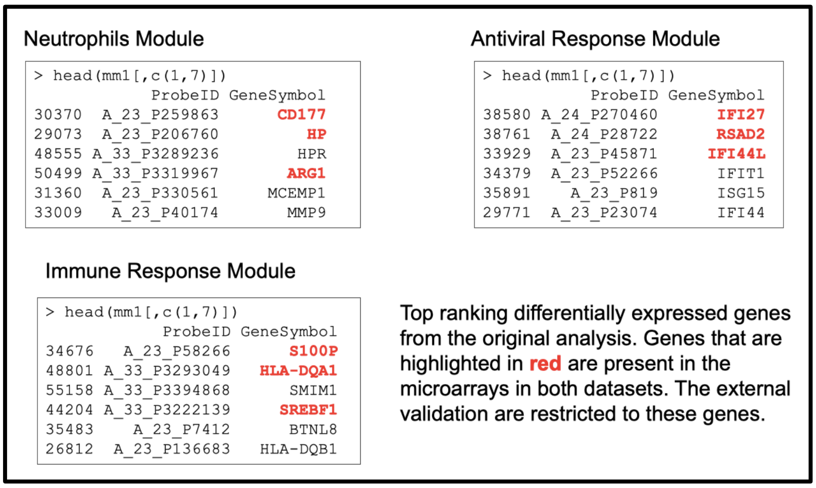

We then proceeded to evaluate whether these genes could accurately discriminate between the "severity 1" and "severity 3" groups in the external dataset (equivalence to "moderate vs. severe" in the original study). The findings are presented in **Supplementary Figure 10**.

RT-PCR and flow cytometry.

RT-PCR: Representative genes were selected from three main modules; *CD177* (neutrophils module), *HLADRA* (immune response module) and *IFI27* (antiviral module). Amplification for CD177 (assay ID: Hs00360669_m1- FAM) and HLADRA (assay ID: Hs00219575_m1- FAM) was performed with 4µl of cDNA in a total volume of 10µl per reaction on a CFX384 (Bio-Rad, Hercules, CA, USA), using the TaqMan gene expression Master Mix (Thermo Fisher Scientific, Australia). The thermal cycle conditions were 95°C for 10 min, 40 cycles of 95°C for 15 s and 60°C for 60 s. Each run included a negative control without RNA template to assess the specificity of the reaction. All assays were done in duplicates and GAPDH (Hs99999905_m1-VIC) was used as the endogenous control. Amplification for IFI27 was performed on Corbett Rotor- Gene 6000 platform (Qiagen) using SYBR green protocol (Life Technologies, Australia) with primer pairs for IFI27 (Fwd: ACCTCATCAGCAGTGACCAGT and Rev: ACATCATCTTGGCTGCTATGG) and GAPDH (Fwd: ACGCATTTGGTCGTATTGGG and Rev: TGATTTTGGAGGGATCTCGC) (Sigma- Aldrich, Australia). The thermal cycle conditions were 95°C for 10 min, 40 cycles of 95°C for 15 s and 60°C for 1 min. Assessment of the specificity of the amplified product was achieved by performing a melting curve analysis.

Flow Cytometry: Peripheral blood of healthy donors and patients with suspicion of influenza was used for evaluation of CD177 expression on granulocytes. Blood samples (100µl) were incubated with anti-CD177 antibody (PE; Exbio, Czech Republic, Clone: MEM-166) for 30min at 4°C in dark. After incubation, lysing solution (1ml of 1X BD FACS™ lysing solution, BD Bioscience, USA) was added for 15min (RT, dark) and finally unbound antibody and lysed erythrocytes were washed out with PBS (5min/21°C/350g). Cell pellet was resuspended in 300µl of PBS and samples were measured immediately using FACS ARIA Fusion flow cytometer (BD Bioscience). Analysis was performed in FlowJo software. Percentage of CD117 positive granulocytes and median of flourescence intensity (MFI) was determined using followed gating strategy: SSC/FCS – cell debris exclusion; SSC/FCS (from previous gated population without debris) for granulocytes selection, SSC/CD177-PE for final evaluation of CD177 expression.

Coinfection patients

The presence of bacterial co-infection might have affected the transcriptomics findings, given the known role of neutrophils in bacterial infection. In this study, seven patients were suspected to have bacterial infection, but only two had positive microbiological confirmation (staphylococcus and streptococcus). The other five patients were suspected to have developed hospital-acquired co-infection during the later course of their illness, although no bacterial pathogens were ever isolated from these five patients. Importantly, the bacterial co-infection was not suspected in these five patients upon enrolment into the study: this was at the time of blood sample collection for transcriptomics profiling (first 24 hours). Thus, the potential impact of bacterial co-infection on transcriptomics findings is likely to be minimal.

SUPPLEMENTARY DISCUSSION

The changes observed in the cell cycle pathways may provide insight into host-virus interaction. Influenza viruses are known to interfere with host cell cycle pathways and in particular, to induce cell cycle arrest at the G0/G1 phase. Previous virology studies have shown that influenza virus induced these changes to provide a more favourable condition for viral protein accumulation and replication.[1,2,3] Indeed, in an earlier study, we found that severe influenza infection was associated with significant changes in host cell cycle pathways, including delayed progression from G0 to G1 phase and dysregulated cell cycle control. [4] The study described here has replicated these earlier findings. A recent study in H7N9 patients also reported a similar findings.[5] Collectively, these findings suggest that host cell cycle pathways may be a useful proxy to help gain insight into host-virus interaction.

Our findings agree with recent studies in humans, all of which identified a strong association between circulating neutrophils and disease severity, especially in patients who presented early (<7 days).[6, 5,7] However, in a subgroup of patients who presented late (>13 days), one study found that monocytes had a greater role than neutrophils in determining infection severity.[7] The differential contribution of neutrophils/monocytes could be related to different patient selection between that study (late presenters) and other studies (early presenters). This difference reveals yet another level of complexity that may have implication for host-directed therapy. Further studies on patient subgroups are clearly needed to clarify this issue.

In this study, patients with severe influenza had significantly higher morbidity and mortality, despite being more likely to be treated with antiviral drugs (91% of patients in the severe group received antivirals compared with 5% in the moderate group). This finding suggests that disease severity in these patients might be more strongly influenced by host factors than viral factors. However, we did not measure viral loads in patients' respiratory epithelia (many patients had received prior antiviral therapy, which makes virus quantification unreliable). This lack of data on viral loads represents a major limitation of this study. Without viral load data, it is difficult to know whether the upregulation of the neutrophils module was a consequence of high viral titers, or if neutrophil activation itself was an independent determinant of disease severity. A recent study by Brandes *et al* specifically sought to address this important question.[8] in that study, the authors discovered a neutrophil-driven inflammatory loop that could directly amplify tissue damage without any contribution from viral load. Furthermore, the authors could reduce lung tissue damage by attenuating neutrophil activity, without changing viral spread in the lung. These findings suggest that the observed neutrophil changes were separate from the effect of viral load. However, these findings were derived from an animal model; we do not know whether similar changes have also occurred in our patients. Clearly, further study is needed to resolve this uncertainty.

Supplementary references

1. Jiang, W. *et al.* Influenza A Virus NS1 Induces G0/G1 Cell Cycle Arrest by Inhibiting the Expression and Activity of RhoA Protein. *Journal of Virology* **87,** 3039–3052 (2013).
2. He, Y. *et al.* Influenza A Virus Replication Induces Cell Cycle Arrest in G0/G1 Phase. *Journal of Virology* **84,** 12832–12840 (2010).
3. Fan, Y. *et al.* Cell Cycle-independent Role of Cyclin D3 in Host Restriction of Influenza Virus Infection. *J. Biol. Chem.* **292,** 5070–5088 (2017).
4. Parnell, G. *et al.* Aberrant Cell Cycle and Apoptotic Changes Characterise Severe Influenza A Infection – A Meta-Analysis of Genomic Signatures in Circulating Leukocytes. *PLoS ONE* **6,** e17186 (2011).
5. Guan, W. *et al.* Clinical Correlations of Transcriptional Profile in Patients Infected With Avian Influenza H7N9 Virus. *The Journal of Infectious Diseases* **368,** 1888–11 (2018).
6. Dunning, J. *et al.* Progression of whole-blood transcriptional signatures from interferon-induced to neutrophil- associated patterns in severe influenza. *Nat. Immunol.* **19,** 625–635 (2018).
7. Cole, S. L. *et al.* M1-like monocytes are a major immunological determinant of severity in previously healthy adults with life-threatening influenza. *JCI Insight* **2,** 1–19 (2017).
8. Brandes, M., Klauschen, F., Kuchen, S. & Germain, R. N. A Systems Analysis Identifies a Feedforward Inflammatory Circuit Leading to Lethal Influenza Infection. *Cell* **154,** 197–212 (2013).
