# Supplementary material for: Neutrophils-related host factors associated with severe disease and fatality in patients with influenza infection
Source: Nat Commun. 2019 Jul 31;10:3422. doi: 10.1038/s41467-019-11249-y (PMC6668409; doi:10.1038/s41467-019-11249-y)
Supplement: Supplementary file 2 — Reporting Summary [file 41467_2019_11249_MOESM2_ESM.pdf]

nature research

Corresponding author(s): Benjamin Tang

Last updated by author(s): May 24, 2019

Reporting Summary

Nature Research wishes to improve the reproducibility of the work that we publish. This form provides structure for consistency and transparency in reporting. For further information on Nature Research policies, see Authors & Referees and the Editorial Policy Checklist.

Statistics

For all statistical analyses, confirm that the following items are present in the figure legend, table legend, main text, or Methods section.

n/a	Confirmed	
☐	☒	The exact sample size (*n*) for each experimental group/condition, given as a discrete number and unit of measurement
☒	☐	A statement on whether measurements were taken from distinct samples or whether the same sample was measured repeatedly
☐	☒	The statistical test(s) used AND whether they are one- or two-sided<br>*Only common tests should be described solely by name; describe more complex techniques in the Methods section.*
☐	☒	A description of all covariates tested
☐	☒	A description of any assumptions or corrections, such as tests of normality and adjustment for multiple comparisons
☐	☒	A full description of the statistical parameters including central tendency (e.g. means) or other basic estimates (e.g. regression coefficient) AND variation (e.g. standard deviation) or associated estimates of uncertainty (e.g. confidence intervals)
☐	☒	For null hypothesis testing, the test statistic (e.g. *F*, *t*, *r*) with confidence intervals, effect sizes, degrees of freedom and *P* value noted<br>*Give P values as exact values whenever suitable.*
☒	☐	For Bayesian analysis, information on the choice of priors and Markov chain Monte Carlo settings
☒	☐	For hierarchical and complex designs, identification of the appropriate level for tests and full reporting of outcomes
☒	☐	Estimates of effect sizes (e.g. Cohen's *d*, Pearson's *r*), indicating how they were calculated

Our web collection on statistics for biologists contains articles on many of the points above.

Software and code

Policy information about availability of computer code

Data collection	Not applicable
Data analysis	Codes and derived data used for the analysis of the microarray expression (normalized data matrix, derived data, PCA, WGCNA, heatmaps, volcano plots, DCQ analysis, boxplots for gene expression, scatter plots, boxplots for module eigengenes, VENN diagrams for modules) is publicly available at doi 10.22000/152 [https://www.doi.org/].

For manuscripts utilizing custom algorithms or software that are central to the research but not yet described in published literature, software must be made available to editors/reviewers. We strongly encourage code deposition in a community repository (e.g. GitHub). See the Nature Research guidelines for submitting code & software for further information.

Data

Policy information about availability of data

All manuscripts must include a data availability statement. This statement should provide the following information, where applicable:

- Accession codes, unique identifiers, or web links for publicly available datasets
- A list of figures that have associated raw data
- A description of any restrictions on data availability

The full dataset that supports the findings has been deposited at the National Centre for Biotechnology Information Gene Expression Omnibus [https://www.ncbi.nlm.nih.gov/geo/]. Access to this dataset is publicly available at GEO accession number GSE101702. Additional data is available upon request by contacting the corresponding author: benjamin.tang@sydney.edu.au.

Field-specific reporting

Please select the one below that is the best fit for your research. If you are not sure, read the appropriate sections before making your selection.

☒ Life sciences ☐ Behavioural & social sciences ☐ Ecological, evolutionary & environmental sciences

For a reference copy of the document with all sections, see nature.com/documents/nr-reporting-summary-flat.pdf

Life sciences study design

All studies must disclose on these points even when the disclosure is negative.

Sample size	The sample size was chosen to have 90% power in detecting differentially expressed genes between severity groups (moderate vs. severe), assuming a false discovery rate of 0.05. Based on gene expression variance and effect size estimated from our previous studies,44, 45 we calculated that a sample size of 46 in each severity group would be required. In total, 750 individuals with flu-like illness were needed to obtain 46 influenza-positive subjects in each severity group (moderate or severe), based on the estimation that (1) only 25% of flu-like illness individuals would be test-positive for influenza virus (estimated from our pilot data), and (2) among those positive for influenza, 13% would develop moderate or severe influenza infection.
Data exclusions	No data were excluded from the analysis.
Replication	The findings were extensively validated and replicated in both independent and external datasets, as described in "cross validation" and "external validation" sections of the manuscript.
Randomization	Not applicable.
Blinding	Not applicable.

Reporting for specific materials, systems and methods

We require information from authors about some types of materials, experimental systems and methods used in many studies. Here, indicate whether each material, system or method listed is relevant to your study. If you are not sure if a list item applies to your research, read the appropriate section before selecting a response.

Materials & experimental systems

n/a	Involved in the study	
☒	☐	Antibodies
☒	☐	Eukaryotic cell lines
☒	☐	Palaeontology
☒	☐	Animals and other organisms
☐	☒	Human research participants
☒	☐	Clinical data

Methods

n/a	Involved in the study	
☒	☐	ChIP-seq
☒	☐	Flow cytometry
☒	☐	MRI-based neuroimaging

Human research participants

Policy information about studies involving human research participants

Population characteristics	This is fully outlined in Table 1 of the manuscript.
Recruitment	This is described in the Method section ("Study design and participants") of the manuscript.
Ethics oversight	The study has been approved by the institutional ethics review board.

Note that full information on the approval of the study protocol must also be provided in the manuscript.
