# Supplementary material for: Neutrophils-related host factors associated with severe disease and fatality in patients with influenza infection
Source: Nat Commun. 2019 Jul 31;10:3422. doi: 10.1038/s41467-019-11249-y (PMC6668409; doi:10.1038/s41467-019-11249-y)

Opening Files as Text in Excel

1. Open Excel
2. Click on Data tab from menu
3. Select Open From Text option
4. Navigate to file location. Under Original data type, “Delimited” should be selected as the file type that best describes your data.
5. Click next at bottom of step 1 of 3 window

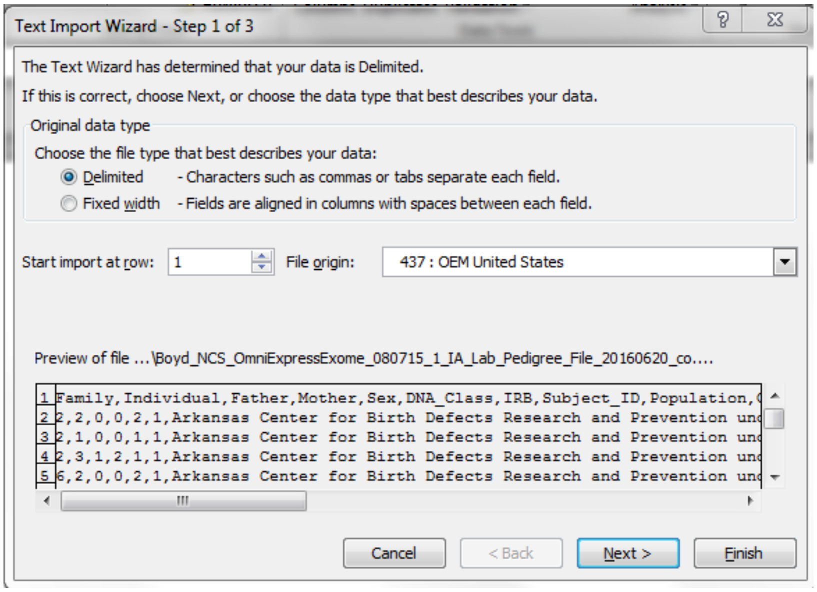

6. In the “Delimiters” section of this window, deselect the “tab” box then select “comma”
7. Click next at bottom of step 2 of 3 window

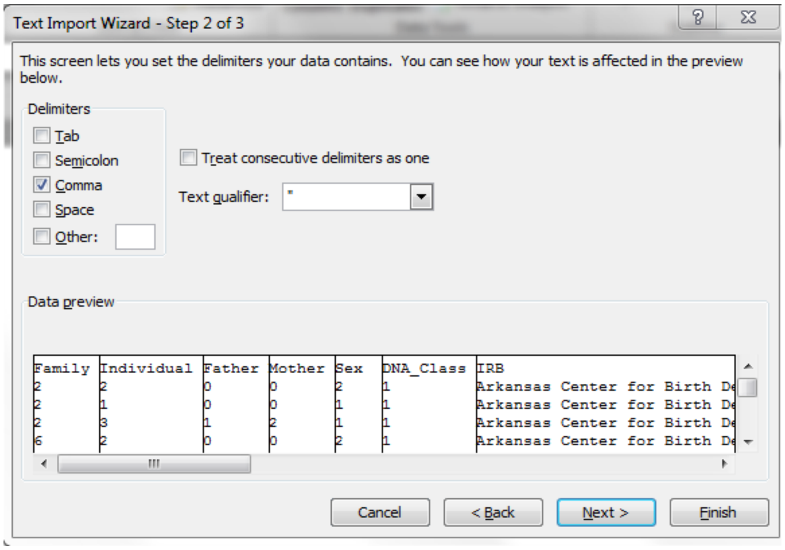

8. Select all columns <u>FIRST</u> - very important!!!
   a. Make sure the first box is highlighted
   b. Drag bottom slide bar to the far right
   c. Hold down shift key and click on the far right column to highlight all

9. Change column data format from “general” to “text”
10. Click finish at bottom of step 3 of 3 window

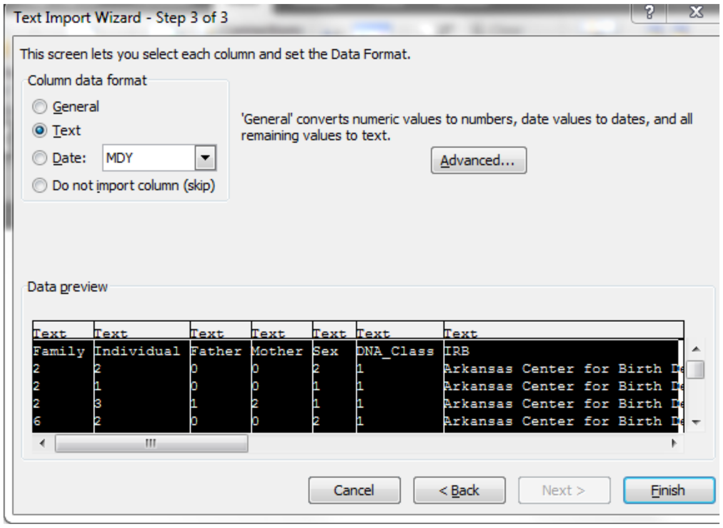

11. Hit okay

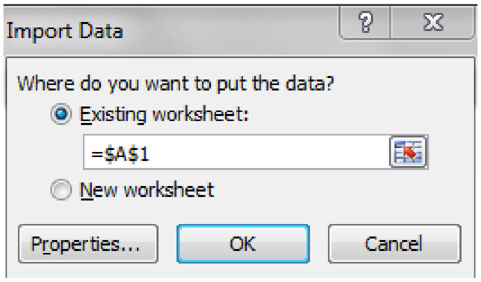

Supplement: Supplementary file 3 — Source Data [file 41467_2019_11249_MOESM3_ESM.zip › File instruction_Supp Fig 8.pdf]
